# Screening for Cyclotides in Sri Lankan Medicinal Plants: Discovery, Characterization, and Bioactivity Screening of Cyclotides from *Geophila repens*

**DOI:** 10.1021/acs.jnatprod.2c00674

**Published:** 2022-12-16

**Authors:** Sanjeevan Rajendran, Blazej Slazak, Supun Mohotti, Taj Muhammad, Adam A. Strömstedt, Małgorzata Kapusta, Emilia Wilmowicz, Ulf Göransson, Chamari M. Hettiarachchi, Sunithi Gunasekera

**Affiliations:** † Phamacognosy, Department of Pharmaceutical Biosciences, Uppsala University, Biomedical Centre, SE 75124 Uppsala, Sweden; ‡ Department of Chemistry, Faculty of Science, University of Colombo, Thurstan Road, Colombo 00300, Sri Lanka; § W. Szafer Institute of Botany of the Polish Academy of Sciences, 46 Lubicz, 31-512 Cracow, Poland; ∇ Department of Plant Cytology and Embryology, Faculty of Biology, University of Gdańsk, 59 Wita Stwosza, 80-308 Gdańsk, Poland; ∥ Faculty of Biological and Veterinary Sciences, Nicolaus Copernicus University, 1 Lwowska Street, 87-100 Toruń, Poland

## Abstract

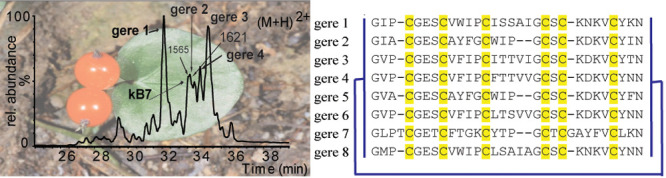

Cyclotides are an intriguing class of structurally stable circular miniproteins of plant origin with numerous potential pharmaceutical and agricultural applications. To investigate the occurrence of cyclotides in Sri Lankan flora, 50 medicinal plants were screened, leading to the identification of a suite of new cyclotides from *Geophila repens* of the family Rubiaceae. Cycloviolacin O2-like (cyO2-like) gere 1 and the known cyclotide kalata B7 (kB7) were among the cyclotides characterized at the peptide and/or transcript level together with several putative enzymes, likely involved in cyclotide biosynthesis. Five of the most abundant cyclotides were isolated, sequenced, structurally characterized, and screened in antimicrobial and cytotoxicity assays. All gere cyclotides showed cytotoxicity (IC_50_ of 2.0–10.2 μM), but only gere 1 inhibited standard microbial strains at a minimum inhibitory concentration of 4–16 μM. As shown by immunohistochemistry, large quantities of the cyclotides were localized in the epidermis of the leaves and petioles of *G. repens*. Taken together with the cytotoxicity and membrane permeabilizing activities, this implicates gere cyclotides as potential plant defense molecules. The presence of cyO2-like gere 1 in a plant in the Rubiaceae supports the notion that phylogenetically distant plants may have coevolved to express similar cytotoxic cyclotides for a specific functional role, most likely involving host defense.

Cyclotides are the largest family of cysteine-rich cyclic peptides in plants, typically biosynthesized as around 28–37 amino acids in size, and thus often referred to as a class of “plant miniproteins”.^1^ A head-to-tail cyclized backbone in combination with a knotted arrangement of three disulfide bonds has given rise to the signature motif of cyclotides, the cyclic cystine knot (CCK).^1^ The CCK motif renders the cyclotides as resistant to thermal, chemical, and enzymatic treatments.^2^ Rubiaceae, Solanaceae, Fabaceae, Cucurbitaceae, and Violaceae are the five major plant families known to bear cyclotides from which more than 400 cyclotide sequences have been characterized to date.^3,4^ Although cyclotides exhibit a wide range of biological activities such as uterotonic,^5^ anti-HIV,^6^ and hemolytic activities,^7^ it is postulated that their natural physiological role in plants is their ability to kill and/or deter insects and pathogens. In support of this view, a range of bioactivities presumably linked to plant defense, including insect growth inhibitory,^8^ antibacterial,^9,10^ antifungal,^10^ and antifouling^11^ activities, have been reported. However, apart from directly utilizing cyclotides for their inherent bioactivities, another main motivation for new cyclotide discovery has been to expand the existing library of ultrastable scaffolds available at hand for peptide re-engineering applications. Termed “epitope grafting”, this concept allows unstable bioactive epitopes to be incorporated successfully within the stable cyclotide framework, as a strategy to impart stability to epitopes of interest. The drug design potential of such “grafted cyclotides” has been successfully demonstrated in the therapeutic areas of pro-angiogenesis,^12^ anti-angiogenesis,^13^ microbial infections,^14^ chronic pain, cancer,^15,16^ and inflammatory management.^17^ Oral absorption^17^ and in vivo efficacy of the grafted cyclotides were prominent in several examples, warranting their further development as therapeutic agents.^14,15^

Cyclotides are grouped into two main subfamilies, Möbius and bracelet, based on the presence or absence of a conserved *cis*-Pro residue in loop 5, which results in a conceptual 180° twist in the backbone (Figure 1).^1^ Aside from this topological feature that is unique to the Möbius subfamily but absent in the other, the two cyclotide subfamilies are further distinguished by variations in the size of intercysteine loops and their sequence. The bracelet form is the largest of all cyclotide subfamilies, containing the most active cyclotides reported, which typically have a higher net positive charge due to the presence of positively charged residues in two of the intercysteine loops (loops 5 and 6). Another distinct feature that demarcates the two subfamilies is the distribution of their hydrophobic residues. Thus, in Möbius and bracelet cyclotides, different loops combine to form a large, surface-exposed, hydrophobic patch. A third subfamily, known as trypsin inhibitors, share very low sequence homologies with the Möbius and bracelet subfamilies but are more similar to linear trypsin inhibitors found in squash plants.^18^ However, because they share the CCK motif and common features in their biosynthesis, they are also categorized as cyclotides.

**Figure 1:**
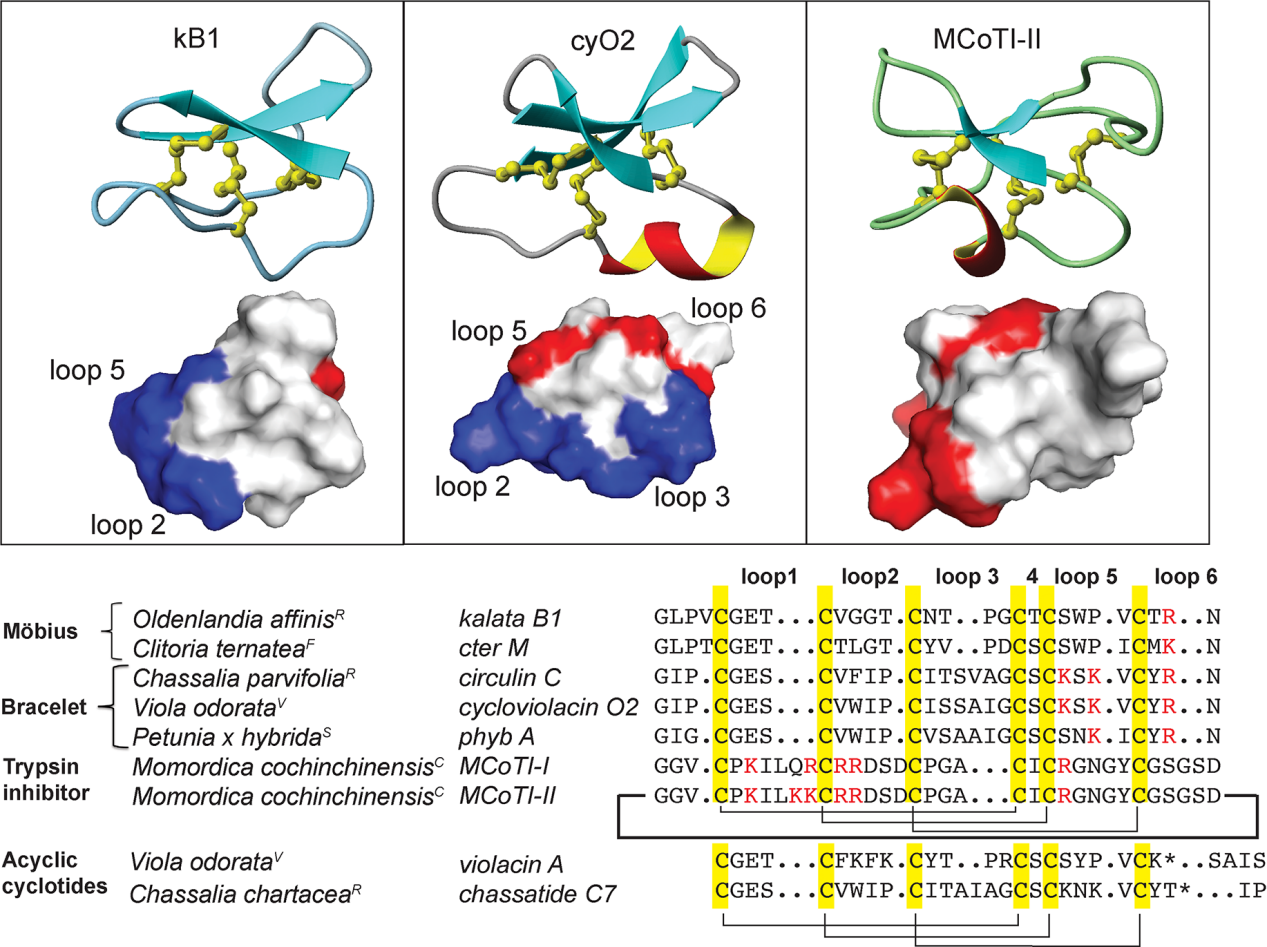
Prototypical cyclotide sequences and structures from the three subfamilies. Three prototypical cyclotides from the three subfamilies kalata B1 (1NB1), cycloviolacin O2 (2KNM), and *Momordica cochinchinensis* II (1IB9) and their surface diagrams are shown on the top panel. For bracelet and Möbius cyclotides, different loops combine to form the hydrophobic patch (highlighted in blue) that is involved in the membrane-mediated mode of action. Residues that are positively charged are highlighted in red. The hydrophilic trypsin inhibitor MCoTI-II acts via another mode of action, entering cells via micropinocytosis. Selected cyclotides and acyclotide sequences from the five plant families (^*R*^Rubiaceae, ^*V*^Violaceae, ^*C*^Cucurbitaceae, ^*F*^Fabaceae, ^*S*^Solanaceae) representative of the three cyclotide subfamilies are shown below. Cysteines are highlighted in yellow, and the corresponding cysteines linked by disulfides that form the cyclic cystine knot (CCK) are indicated. The acyclotides contain a stop codon in the gene for the peptide that prevents translation beyond the residue highlighted by an asterisk (*). The cyclic backbone in cyclotides is depicted by the bold-lined N to C connection that is lacking in the acyclotides.

Of all the plant families screened for cyclotides, every species of the Violaceae appears to produce cyclotides. In contrast, only about ∼10% of the Rubiaceae species screened contain cyclotides.^3,19,20^ By screening >200 Rubiaceae species, Gruber et al. identified *Geophila repens* (*G. repens*) as one of the 22 plants containing cyclotides.^20^ However, despite obtaining initial confirmation for the presence of cyclotide-like masses, individual cyclotides were neither isolated nor sequenced. *Geophila* is a genus of the Rubiaceae family containing about 30 species. Commonly found in tropical Asia and Africa, *G. repens* grows as a creeping evergreen herb, reaching up to around 4 cm height and flowering during March to September.^21^ As a remedy to treat coughs, a decoction of boiled *G. repens* is used in Sri Lankan traditional medicine.^22^ The fruits of *G. repens* are also used as an antifungal agent.^23^ As there is preliminary evidence supporting the occurrence of cyclotides as well as local knowledge of ethnomedicinal usage, *G. repens* was selected for detailed investigation of its cyclotide content. Herein the aim was to characterize new cyclotides from *G. repens* and to evaluate their bioactivity, specifically their cytotoxicity to mammalian cells and antimicrobial activity against bacteria and a fungus.

A few years after the initial discovery of cyclotides, their cytotoxic and membrane-disrupting properties became evident.^24^ Since then, the cytotoxic potential of cyclotides has been explored via a number of strategies. First, their direct ability to kill cancer cells has been evaluated, with to date nearly 70 cyclotides having been tested against various cancer cell lines to establish their IC_50_ values.^25^ In a second strategy, the cyclotide scaffold has been employed as a stabilizing template to graft epitopes implicated in potential anticancer applications, with both a therapeutic and/or diagnostic focus.^13,26,27^ Third, the concurrent use of cyclotides with anticancer drugs as a means to sensitize cancer cells is being evaluated.^28^ One setback in this regard has been the nonspecific cytotoxicity of cyclotides, because even at low micromolar concentrations, most cyclotides disrupt membranes in a nonselective manner.^29^ Despite this, interest in the discovery of new cytotoxic cyclotides has continued to rise, in the hope of identifying putative sequences that selectively bind/modulate cancer-specific targets.

Herein are reported the characterization of eight new bracelet-type cyclotides and the presence of one known Möbius-type cyclotide in *G. repens* as a result of using a combination of tools, including de novo MS/MS sequencing, transcriptomics, and NMR analysis. In addition, the characterization of precursor proteins of the new cyclotides and their putative biosynthetic processing enzymes was conducted. The cancel cell line cytotoxic activity of all cyclotides was tested against lymphoma cells, and their antimicrobial activity was investigated, using standard microbial strains as well as *E. coli* membranes. Finally, via immunohistochemical analysis, the location of the most potently cytotoxic cyclotide in the leaves of *G. repens* was established to gain insight into the natural physiological role of this compound class in planta.

## Results and Discussion

### Screening for Cyclotides in Fabaceae, Rubiaceae, and Solanaceae Plants

This project was initiated with the aim of delineating the distribution of cyclotides within traditionally used Sri Lankan medicinal plants. The study then focused on *G. repens*, the only plant confirmed to contain cyclotides. Selected plants were investigated to determine whether cyclotides are the bioactive constituents responsible for anecdotal accounts of plant usage in traditional medicine. Initially, 50 plants including 28 from the Fabaceae, 15 from the Rubiaceae, and seven from the Solanaceae (Table S1, Supporting Information) were selected based on their ethnomedical use and because they belong to plant families known to contain cyclotides. Two of the plant species, *Knoxia zeylanica* and *Wendlandia bicuspidata*, are endemic to Sri Lanka with very limited phytochemical accounts published prior to this study;^22,30^ thus they were also included despite not having any documented usage in traditional medicine.

The crude aqueous extracts obtained by small-scale extraction of the 50 medicinal plants were subjected to RP-HPLC. All chromatographic peaks were investigated manually for the presence of cyclotide-like masses. A parent ion in the 2500–3300 Da range, deconvoluted from the isotopic pattern for the 2+ (∼0.5 Da distance between the isotopic peaks) and 3+ (∼0.33 Da distance between the isotopic peaks) charged states, was used in the initial characterization.^1^

Of the 50 plants screened, only *G. repens* from the family Rubiaceae showed peaks with cyclotide-like masses at late-eluting retention times, between 28 and 36 min (Figure S1, Supporting Information). There were no cyclotide-like masses in the chromatograms for the other plant species investigated belonging to the Fabaceae, Rubiaceae, or Solanaceae. In the Fabaceae alone, 28 plants were screened in total. Although the expression pattern of cyclotides may vary according to biotic and abiotic factors,^31^ there were no reports of even minute amounts of cyclotides found for any given plant. Typically, at least one or a few cyclotides are expressed in high abundance when any cyclotides are present. Thus, it appeared from the analysis conducted that cyclotides were absent from rest of the screened plants aside from *G. repens*.

### Large-Scale Isolation of Cyclotides from *Geophila repens*

**Figure 2:**
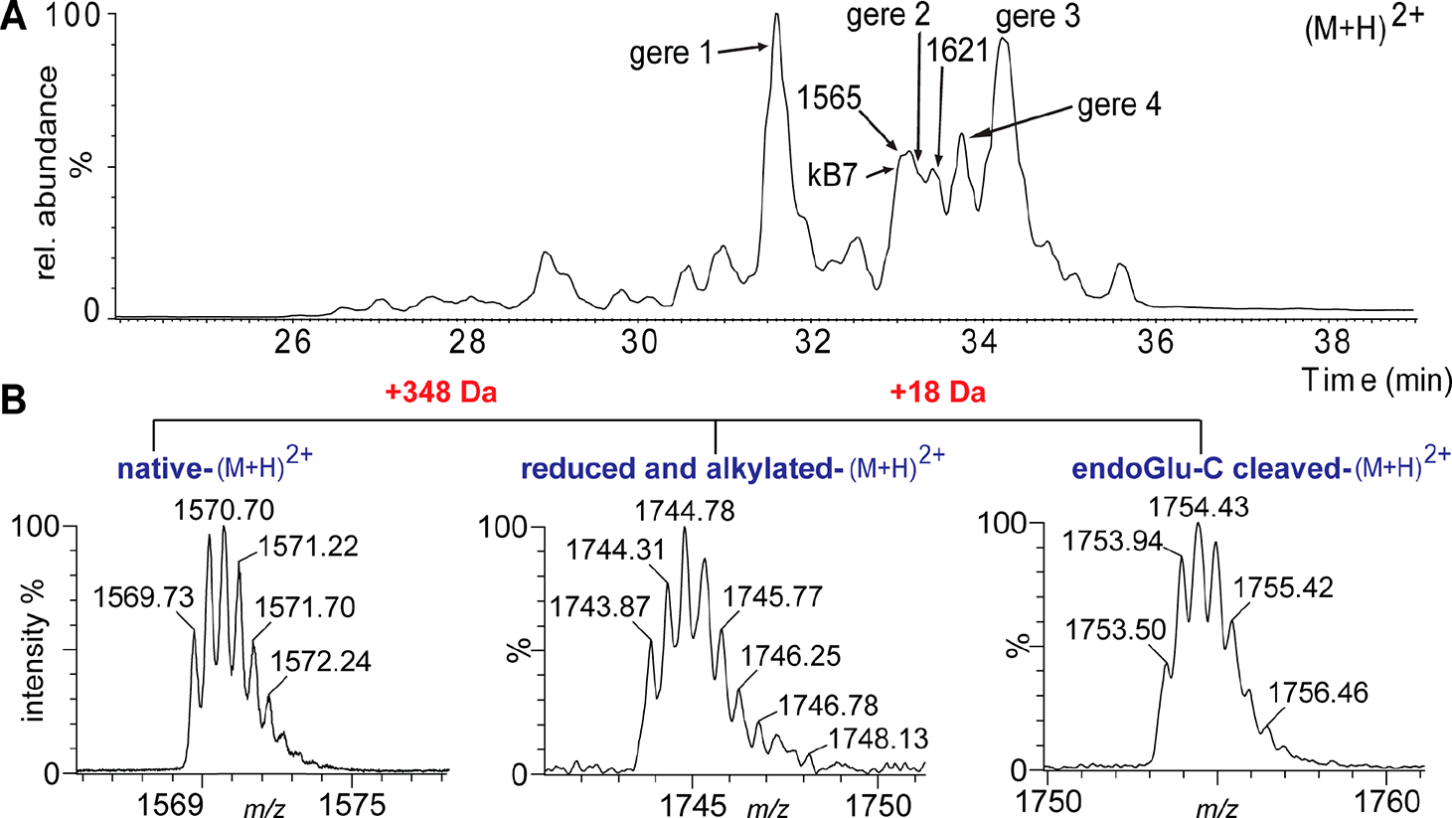
RP-HPLC profile of *G. repens* extract containing putative cyclotides and confirmation of their CCK motif by disulfide reduction and alkylation followed by enzymatic digestion of the cyclic backbone. (A) The late-eluting peaks with molecular weights ranging between 2500 and 3300 Da indicated the presence of cyclotide-like masses, leading to the isolation of the most abundant peptides, geres 1–4 and kB7. (B) Reduction and alkylation of the six cysteines resulted in a mass shift of 348 Da. A further mass increase of +18 Da resulted in endo-GluC digestion at a single conserved Glu.

From 140 g of air-dried plant material, ∼5 to ∼8 mg each of five major cyclotide masses was obtained. The five cyclotides eluted between 35% and 60% of CH_3_CN using RP-HPLC (Figure 2). The peptides with the corresponding monoisotopic masses eluted in the following order of hydrophobicity: *m*/*z* 3137.37 (gere 1) < 3069.27 (kB7) < 2964.20 (gere 2) < 3147.31 (gere 4) < 3114.35 (gere 3) (Figure 2). Fractions obtained from preparative RP-HPLC were further purified to obtain pure cyclotides with >95% purity as assessed by UV (PDA detector at 215, 254, and 280 nm).

### Modification of Cyclotides to Identify Sequences by Mass Spectrometry

Due to the stability of the CCK motif, chemical modification was required to facilitate sequence determination of isolated cyclotides by MS/MS. The cysteines were reduced, alkylated, and converted to carbamidomethyl cysteines. A total increase in mass by 348 (58 for each cysteine × 6) Da confirmed the presence of six cysteines in each peptide (Figure 2). The modified cyclotides eluted earlier than their native counterparts, presumably because the hydrophobic side chains were no longer exposed on the surface. The reduced, alkylated cyclotides were then digested by enzymes to facilitate efficient fragmentation by MS/MS.

Typically, a single product is obtained using endoproteinase GluC (endo-GluC), because cyclotides contain a single conserved Glu residue and endo-GluC selectively cleaves after Glu residues. Fragments of the cyclotide are also obtained by digestion with trypsin, which cleaves after the C-terminus of the positively charged amino acids such as Lys or Arg. Herein, five peptides were subjected to endo-GluC and trypsin cleavage.

**Figure 3:**
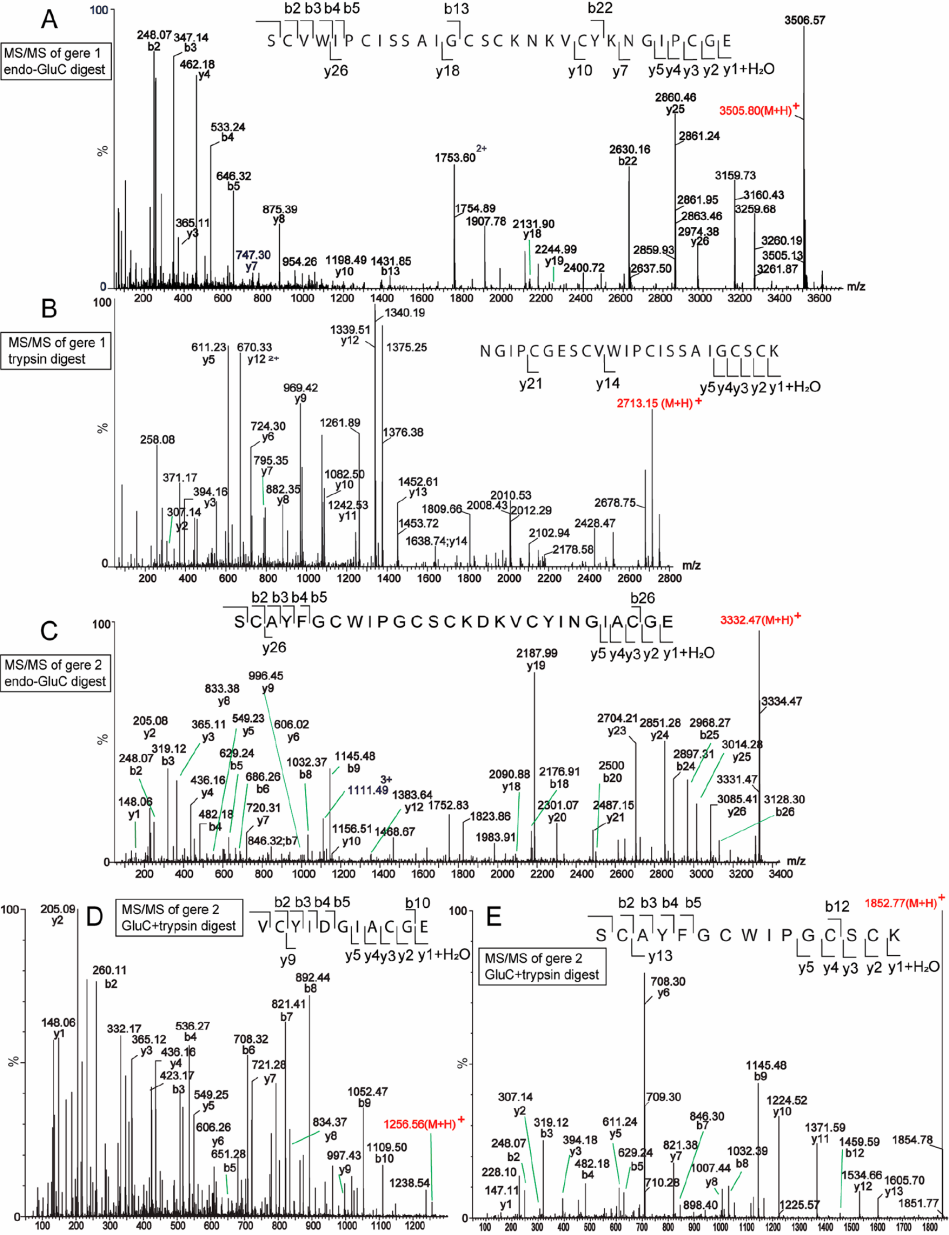
MS/MS sequencing of the two cyclotides found in *G. repens*. (A) Endo-GluC cleaved gere 1 (amino acid sequence GIPCGESCVWIPCISSAIGCSCKNKVCYKN cyclic) after reduction and carbamidomethylation. (B) Trypsin-cleaved gere 1. (C) Endo-GluC cleaved gere 2 (amino acid sequence GIA-CGESCAYFGCWIPGCSCKDKVCYIN cyclic) after reduction and carbamidomethylation. (D) Reduced, alkylated, endo-GluC cleaved, and subsequently trypsin-cleaved gere 2 peptide fragment 1. (E) Reduced, alkylated, endo-GluC cleaved, and subsequently trypsin-cleaved gere 2 peptide fragment 2.

The sequence of gere 1 was determined to be identical to cyO2 except at two positions where corresponding amino acids were substituted, namely, Ser24Asn and Arg29Lys. Upon endo-GluC cleavage, the reduced and alkylated gere 1 (cyO2 like) increased by 18 Da in mass, indicating the presence of a single conserved Glu residue and the cyclic backbone of the peptide. The resulting linear product was subjected to ESIMS and was observed as a triply charged ion, which was selected as the precursor ion for MS/MS fragmentation (Figure 3). The endo-GluC cleaved sample was further cleaved by trypsin, and the resultant fragment SCVWIPCISSAIGCSCK then used as a precursor ion for MS/MS fragmentation (Figure 3). On the basis of the b- and y-ion series annotated in the MS/MS spectrum, the complete sequence of gere 1 was determined. The fragments NK and VCYK resulting from trypsin cleavage could not be detected by MS most likely because of its hydrophilic nature and early elution in the LC-MS. However, the identity of these fragments was later confirmed by NMR spectroscopy and further supported by the presence of the sequence in the transcriptome.

The new cyclotide gere 2 (1484^2+^) yielded one fragment of monoisotopic mass 2968.15 Da upon digestion with endo-GluC. The fragment SCAYFGCWIPGCSCK, with an average mass of 1852.77 Da, could be identified by trypsin cleavage, but the fragment NK could not be detected by MS; however, this was resolved later by NMR analysis. The full sequence was deciphered by the b- and y-ions resulting from MS/MS fragmentation of the precursor ions. Gere 2 is similar in sequence to hyfl J from *Hybanthus floribundus*,^32^ except for a Arg21Lys substitution in loop 5 and a Ile27Phe substitution in loop 6.

A similar de novo sequencing approach was followed for gere 3, gere 4, and kB7, where the reduced and alkylated peptides were subjected to endo-GluC and trypsin digestion. These enzymatic treatments yielded one or more peptide fragments that were subjected to further fragmentation using MS/MS. The MS/MS spectra containing the assigned b- and y-ion series that enabled the elucidation of the peptide sequences are shown in Figure S1, Supporting Information.

Notably, Asn to Asp deamidation could be observed commonly during MS/MS fragmentation and typically as a mixture of both Asn and Asp isoforms in all *G. repens* cyclotides (Table S2, Supporting Information). Deamidation has been noted to occur during enzyme treatment of cyclotides during de novo sequencing (the buffer conditions at pH 8 and 37 °C heating). Additionally, Asn residues in cyclotides can undergo deamidation during storage or enzymatic cleavage.^3^

### NMR Analysis

NMR spectroscopy is a valuable tool to obtain structural data and insight into the stability and chemical folding of cyclotides.^1^ Distinct amino acids have characteristic patterns of spectra, and this in combination with the knowledge of their chemical shifts typically can be helpful to differentiate certain amino acids. Despite this, determination of sequential amino acids in a new cyclotide by NMR-based resonance assignment alone is difficult, without any prior knowledge on the primary sequence. This is because cyclotides have well-defined three-dimensional structures, resulting in many medium- and long-range NOE cross peaks among sequential as well as nonsequential amino acids. Thus, the identification of consecutive amino acids in a peptide chain becomes an arduous task. Additionally, identifying residues corresponding to an AMX spin system (i.e., Cys, Asp, Phe, His, Tyr, Ser) can be ambiguous. However, when complemented with MS/MS sequencing, NMR spectroscopy is a powerful supporting tool for de novo sequencing. In the current study, ^1^H 2D-TOCSY and NOESY experiments facilitated the sequencing as well as the structural comparison of the new *Geophila* peptides.

**Figure 4:**
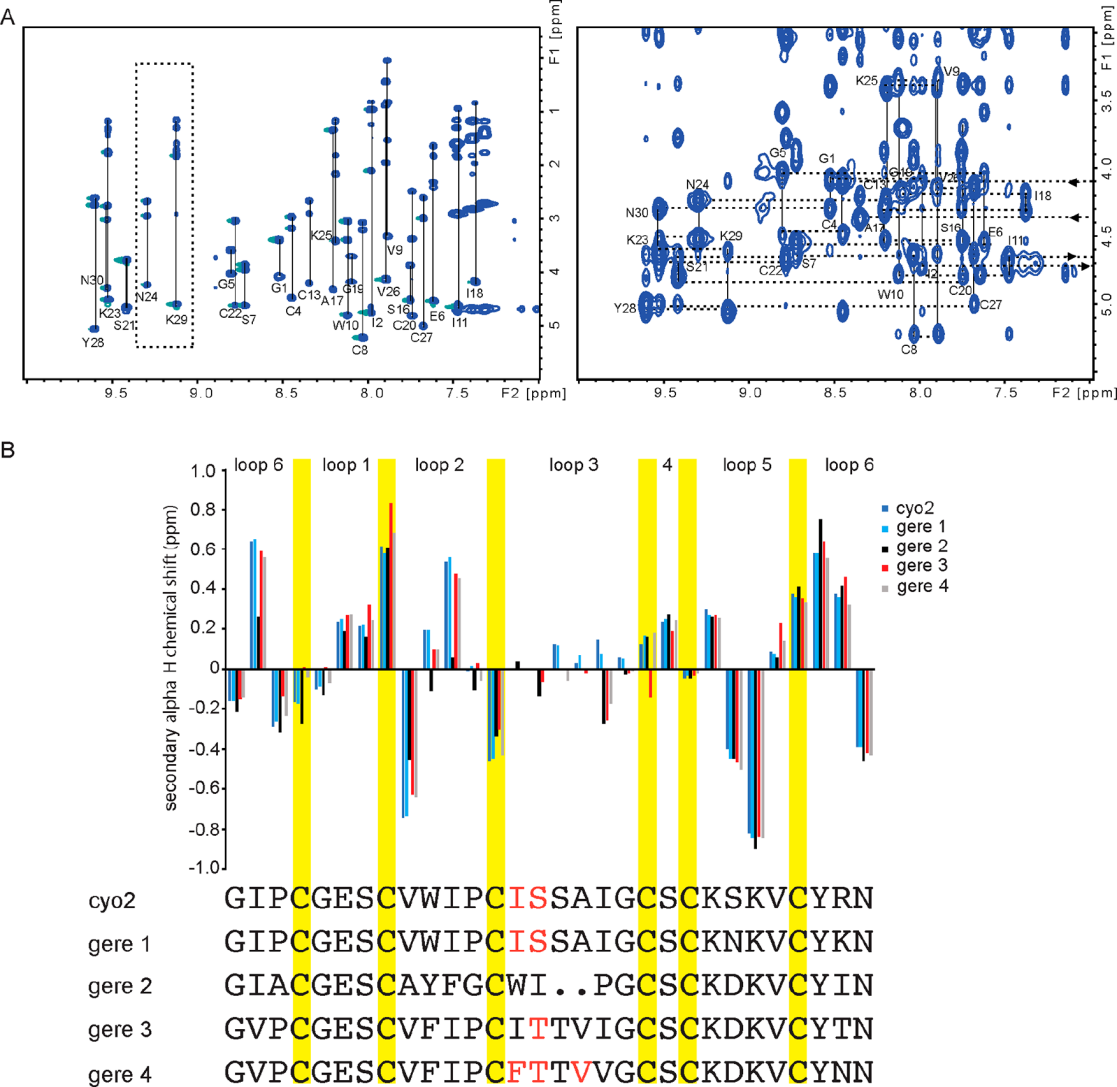
NMR-based structural characterization of the five cyclotides isolated from *G. repens.* (A) TOCSY and NOESY spectra for gere 1. TOCSY spectrum (left) containing amino acid spin systems. Two residues (Asn24 and Lys29) that differ in sequence from cyO2 are highlighted in a box. NOESY spectrum (right) showing the amide region and sequential amino acid connections (“sequential walk”), confirming the head-to-tail cyclic backbone. (B) A comparison of secondary αH proton chemical shifts of *G. repens* cyclotides with cyO2. The similar pattern of secondary αH proton chemical shifts indicates that gere 1–4 cyclotides contain a very similar overall fold to cyO2. Some of the residues in loop 3 were absent in the spectra (highlighted in red), presumably due to unfolding of the typical helix in this region. Gere 2 deviates in secondary αH proton chemical shifts at certain residues (mainly in loop 2 and loop 6), indicating local structural differences.

Comparison of their TOCSY spectra showed that gere 1 was very similar to cyO2 except for the loop 5 region (Figure 4). In comparison to cyO2, the typical amino acid spin pattern for a Ser, which often has β-protons shifted downfield as compared to other AMX spin systems, was absent at position 24 of gere 1. Taken together with the MS data, it was determined that the corresponding position is occupied by an Asn, which was corroborated by the spin system pattern observed by NMR analysis. Similarly, the sequences obtained for peptides gere 2–4 and kB7 by MS/MS sequencing were confirmed by NMR chemical shift assignments of the amino acids based on sequential NOE correlations (Figure S2, Supporting Information). In particular, isobaric residues such as Gln/Lys and Ile/Leu are not distinguishable in MS/MS. These residues were resolved using NMR analysis based on their characteristic spin systems and chemical shift values. In addition, the *cis–trans* conformation of prolines can be observed by NOE cross-peaks of d_αα_ or d_αδ_ for proline (i) and its preceding residue (i-1). Of all gere cyclotides, only kB7 (Möbius-type) contained such a *cis*-pro in loop 5, allowing its characterization as a Möbius cyclotide. The remaining gere cyclotides were devoid of a proline in loop 5, and thus were categorized as bracelet subfamily members.

α-Proton chemical shifts are very sensitive to structural changes and thus can be used for structural comparisons. Herein, the secondary α-proton chemical shifts of the new peptides were compared with those of cyO2. Overall, the secondary α-proton pattern for gere 1–4 followed the same pattern as for cyO2, confirming the presence of the stable CCK motif for all peptides. In particular, gere 1 showed a closely matching and nearly identical secondary structure to cyO2, confirming a very similar structure. Some deviations in secondary α-proton chemical shifts for the loop 2, loop 3, and loop 6 regions of gere 2 could be observed, indicating regions with structural deviations from the counterpart regions of cyO2. These are the regions where amino acids are different between gere 2 and cyO2, and these sequence differences likely lead to local structural perturbations, despite maintaining the overall CCK fold.

### Analysis of Cyclotides, Their Precursor Proteins, and Putative Biosynthetic Processing Enzymes at the Nucleic Acid Level by Transcriptomic Analysis

To aid and complement de novo sequencing, transcriptomic analysis was conducted, which has proven to be a useful technique in cyclotide discovery.^19,33^ Despite several attempts, getting a quality RNA sample from the Sri Lankan *G. repens* plant containing cyclotides was not successful, due to RNA degradation during isolation and transport for sequencing. Serendipitously, the same species was identified growing in Uppsala Botanical Garden, from which RNA was isolated and the transcriptome was assembled. The peptides were identified by tBLASTn searches using known cyclotide precursor sequences as queries. Contigs from resulting homologues were translated and annotated based on published cyclotide precursors. This approach led to the identification of five cyclotide precursors at the transcriptome level, including one sequence containing a mature cyclotide domain-matching gere 1 cyclotide identified at the peptide level. Although from a different geographical location from the Sri Lankan plant used for peptide extraction, gere 1 transcript was found in the plant from Sweden, supporting its position as a key peptide of functional significance. Notably, corresponding cyclotides for the four other transcripts (gere 5–8) were absent at the peptide level (Table 1). Earlier studies have suggested that one plant species can express up to ∼15–60 cyclotides.^31^ Strikingly, in the analysis conducted only five cyclotide transcripts were identified in *G. repens*. It may be speculated that cyclotide genes may remain dormant when the production of cyclotides for host defense is not urgent. This may be the case when a tropical plant does not encounter its natural predators and is growing under more secure conditions like in a temperate botanical garden. In fact, the cyclotide production patterns differed greatly in extracts obtained from the specimen from the botanical garden and from Sri Lanka (Figure S1, Supporting Information). Additionally, the changes in production patterns of cyclotides can be attributed to factors such as variations in the type of soil, the availability of sunlight, altitude, water, humidity, and temperature, which could also vary between different geographical locations.^34^

**Table 1:**
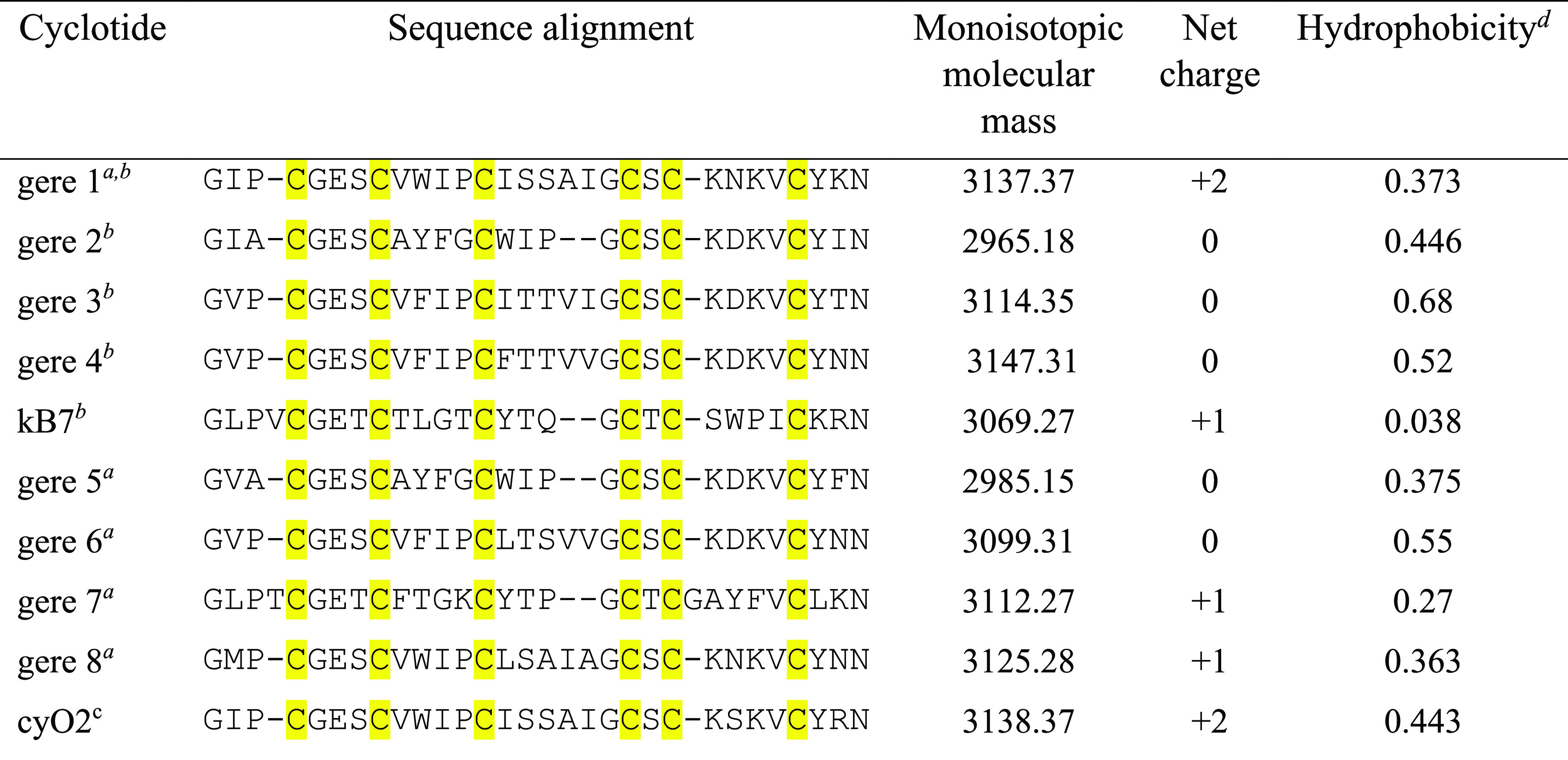
New Cyclotides Characterized from *G. repens*

a Predicted from transcripts.

b Detected at peptide level and sequenced with MS/MS.

c cyO2 sequence is included for comparison (http://www.cybase.org.au).

d Grand average of hydropathicity was calculated using expasy protein parameter calculation tool (https://web.expasy.org/protparam/).

**Figure 5:**
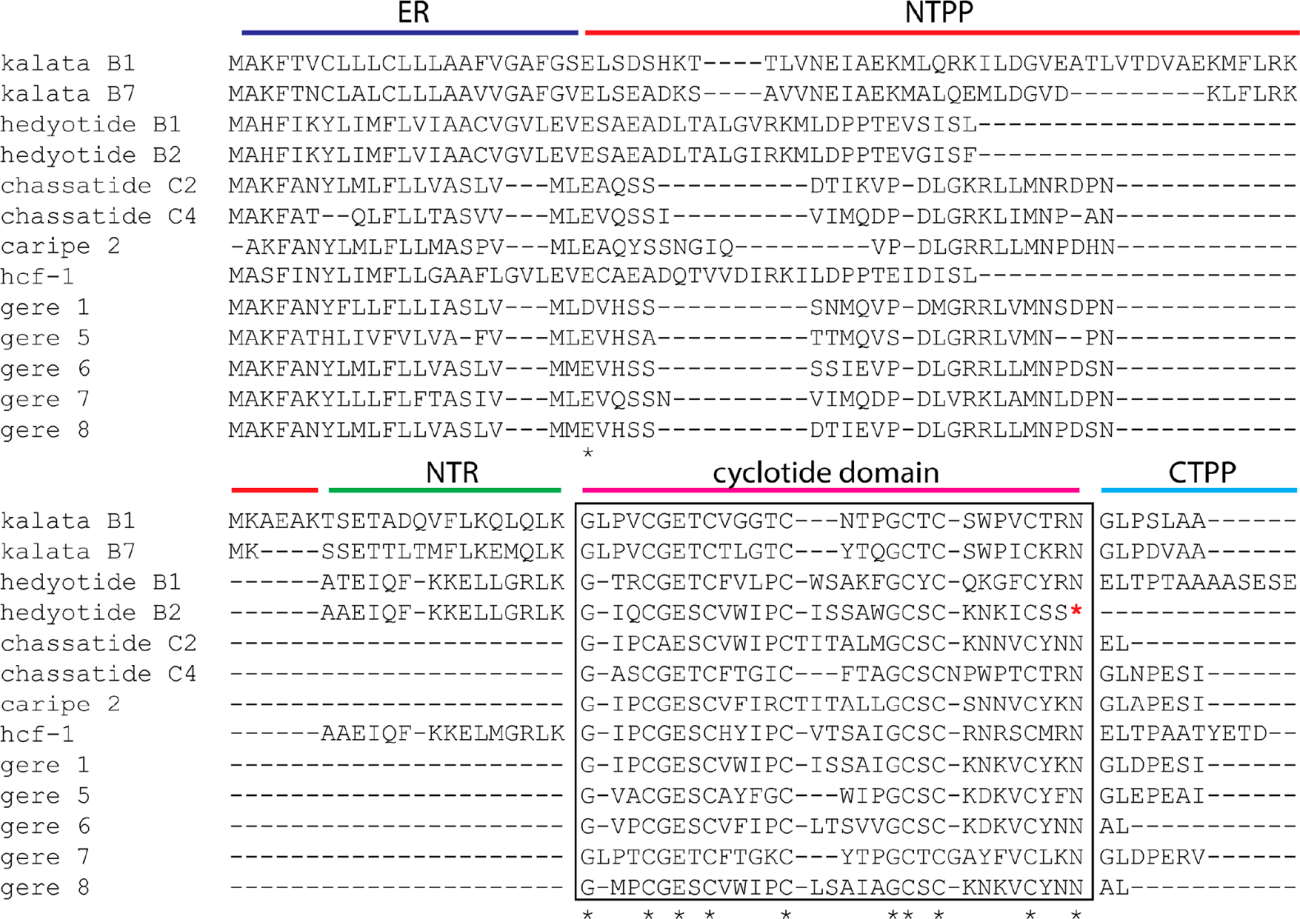
Multiple sequence alignment of gere cyclotide precursors with known examples of Rubiaceae cyclotide precursors. kB1 (*Oldenlandia affinis*), kB7 (*Oldenlandia affinis*), hedyotide B1 (*Hedyotis biflora*), hedyotide B2 (*Hedyotis biflora*), chassatide C2 (*Chassalia chartacea*), chassatide C4 (*Chassalia chartacea*), caripe 2 (*Carapichea ipecacuanha*), and hcf-1 (*Hedyotis centrathoides*). The putative ER domain is highlighted in dark blue, with the NTPP domain in red, the NTR domain in green, the cyclotide domain in pink, and the CTPP domain in cyan. A stop codon is found in the RNA, and consequently the peptide is translated as an acyclotide in hedyotide B2, as highlighted by an asterisk (*).

To date, cyclotide precursor sequences have been reported from the Rubiaceae genera *Oldenlandia*,^35^*Hedyotis*,^20^*Chassalia*,^36^ and *Carapichea*(19) by PCR-based cDNA library construction (Figure 5). Rubiaceae cyclotide precursors are generally organized with an ER signal, a propeptide domain, an N-terminal repeat domain, a mature cyclotide domain, and a C-terminal signal peptide. Within the Rubiaceae, *Chassalia* precursors are the only exception identified so far with reduced NTPP, NTR, and CTPP regions.^36^ Notably, the *Geophila* cyclotide precursors identified herein also have very shortened precursors, with shortened NTPP and NTR regions similar to *Chassalia* cyclotide precursors. This is not surprising given the close phylogenetic relationship between the two genera. Different functional roles are suggested for the NTR, such as folding and vacuolar targeting,^37^ but the *Geophila* precursor architecture suggests that the NTR region may not play an essential role in cyclotide biosynthesis. It is speculated that *Chassalia* plants express shortened *Chassatide* precursors as part of a shade-tolerant mechanism to adapt to low-light conditions and energy conservation, a theory built on the observation that *Chassalia* plants grow under shady conditions.^36^ It is interesting to note that *Geophila* is also a shade-preferring plant found under tree canopies in its natural habitat where sunlight is likely a limiting factor. However, this highly speculative hypothesis needs to be validated by studying the genetic arrangement of additional cyclotide members growing under similar environmental conditions.

An N-terminal Gly and a C-terminal Asn are conserved in the mature cyclotide domain of all gere cyclotide precursors. Next to the N-terminal processing site, *Geophila* precursors resemble Violaceae cyclotide precursors by typically having a conserved Asn (as the C-terminal residue of NTR). P1′ and P2′ residues, trailing the cyclic peptide domains, consist of a small amino acid (Gly or Ala) and a conserved Leu, respectively. The latter feature is more aligned with Rubiaceae precursors.^38^ Notably, no acyclotides were identified in *Geophila* precursors.

The biosynthesis pathway of cyclotides is not yet fully deciphered. However, enzymes such as protein disulfide isomerase (PDIs) and asparaginyl endopeptidases (AEPs) presumably facilitate folding and cyclization of cyclotides in vivo. In forming the mature cyclized backbone, cyclotide precursors must undergo an N- and C-terminal cleavage by a protease-like enzyme, followed by N- to C-terminal ligation by a ligase-type enzyme.^38,39^ Although AEPs commonly function as proteases, the presence of cyclizing enzymes capable of forming the head-to-tail backbone of cyclotides, with a predominant “ligase” character, has been verified in functional studies, mostly from Violaceae spp. and in one example of a Rubiaceae spp.^40,41^ In the current study, several transcripts for *Geophila* enzymes, with a matching AEP catalytic region, were identified. Of these, only GrAEP3 showed >72% sequence similarity to OaAEP1_b_ from the Rubiaceae family.^40^ (For a detailed comparison of the *Geophila* AEP catalytic regions, refer to Figure S3, Table S3, Supporting Information) Notably, the remaining three *Geophila* AEPs were more similar to Violaceae AEPs. The first two enzymes, GrAEP1 and GrAEP2, share at least a >75% sequence identity with *Viola yedoensis* VyAEP4.^41^ The third enzyme, GrAEP4, is more similar to *V. yedoensis* VyAEP1 (>65% sequence similarity).

Another conspicuous feature in gere cyclotide precursors was a conserved Asn found at both the N- and C-terminal processing sites. This is supportive of gere AEPs likely being involved in both N- and/or C-terminal processing. Such bifunctional enzymes are not entirely new to cyclotide-bearing plants. In *Momordica cochinchinensis* AEP2, MCoAEP2 is capable of N-terminal excision and C-terminal cyclization of cyclotide precursors in vitro.^42^ However, to which extent each gere AEP is involved in cyclotide biosynthesis and their precise physiological role is yet to be shown by future functional studies. Aside from AEPs, our group found also three transcripts matching PDI enzymes likely involved in gere cyclotide oxidative folding. Two of these peptides, *Gr*PDI 1 and *Gr*PDI 2, showed >82% and >70% sequence similarity to *Oa*PDI, respectively.^39^ The other PDI enzyme, GrPDI 3, showed around 65% sequence similarity to *Viola betonicifolia* VbPDI 2.^33^

### Antimicrobial Activity

Cyclotides isolated from *G. repens* were subjected to a microdilution assay on 96-well plates to determine minimum inhibitory concentration (MIC values), according to a method devised to minimize the activity-inhibiting presence of rich growth media.^43^ For these experiments, bacterial strains of *E. coli*, *P. aeruginosa*, and *S. aureus* and the fungal strain of *C. albicans* were used. The MIC was taken as the lowest concentration of a cyclotide where the visible growth of microbes was inhibited, i.e., 100% of cell death. All cyclotides were inactive against the panel of microorganisms used at the highest tested concentration of 32.5 μM, except gere 1, which showed significant activity against both bacteria and fungi. Notably, the highest activity of gere 1 was seen against *E. coli* with a MIC of 4.0 μM (Table 2).

**Table 2:** MIC of Cyclotides Extracted against *E. coli*, *P. aeruginosa*, *S. aureus*, and *C. albicans* and Cytotoxic Activity in the FMCA Using the Human Lymphoma Cell Line U-937 GTB

antimicrobial activity, MIC (μM)					cytotoxicity, IC_50_ (μM)
cyclotide	*Escherichia coli*	*Pseudomonas aeruginosa*	*Staphylococcus aureus*	*Candida albicans*	lymphoma U937
gere 1	4.0	7.9	>10	>10	2.0
gere 2	>10	>10	>10	>10	6.1
gere 3	>10	>10	>10	>10	2.2
gere 4	>10	>10	>10	>10	2.6
kB7	>10	>10	>10	>10	>10
cyO2^a^	>10	10	>10	>10	0.26–1.8
kB7^a^	>10	1.25	>10	>10	>10

a Previously reported antimicrobial activity^43^ and cytotoxicity^44^ for the known cyclotides cyO2 and kB7 were included for comparison.

Cyclotide antibacterial and anticandidal properties are well-debated topics by various research groups detecting no or minimal activity for identical peptides on similar microbes using different methods and protocols.^43^ The antimicrobial peptide (AMP)-inhibiting influence of rich growth media seems to play a part in the divergent reports, where cyclotides seem particularly susceptible and lose antimicrobial activity. However, more robust and potent activity is found within the cycloviolacin cyclotides, which exhibit activity against a broad spectrum of bacteria, particularly Gram-negative species,^43^ and against *Candida* species.^9,43^ In this study, a cyO2-like gere 1 was identified only differing in two functionally similar amino acid substitutions from cyO2. The roughly similar level of antimicrobial activity between cyO2 and gere 1 is reasonable given their sequence similarities, taking into account that the separate 2-fold microdilution series are not identical and thus introduce an artificial impression of a difference in MIC. The only assay where gere 1 and cyO2 differed significantly was against *E. coli*, where gere 1 proved to be 5-fold more active. A different situation was seen with kB7, which did not exhibit activity below 32.5 μM in this study, as supported by being active only at 2 mM in radial diffusion assays previously.^45^ Notably, kB7 was active at 1.25 μM on *P. aeruginosa* in a study with similar experimental conditions to that used presently.^43^ In this instance, it was speculated that the antibacterial activity could be connected to external exposure of PE lipids during bacterial division and growth. As the assay is performed in buffer, slight deviations in experimental handling, such as delays during transfer from culture to the actual assay, might affect the resultant activity.

The importance of the antimicrobial activity of cyclotides has not yet been studied in a phytopathogenic context, using methods that reflect the specific conditions present in plant tissues. However, cycloviolacin peptides are shown to be active against plant fungi.^10^ CyO2, in particular, and to a lesser extent also kB1 and kB2 are toxic to soil bacteria but also to other plants, which might make them allelopathic agents for their host.^46^ Thus, a way forward in elucidating the potential antibacterial modality for cyclotides will be to investigate their effects at more physiologically relevant and environmentally dynamic conditions concerning host plants. Aside from these, grafted cyclotides have also shown promise in the antibacterial field where the scaffolds incorporated with antimicrobial sequences have given low micromolar in vivo efficacy, especially against clinical isolates.^14^

### Liposome Assay Results

**Figure 6:**
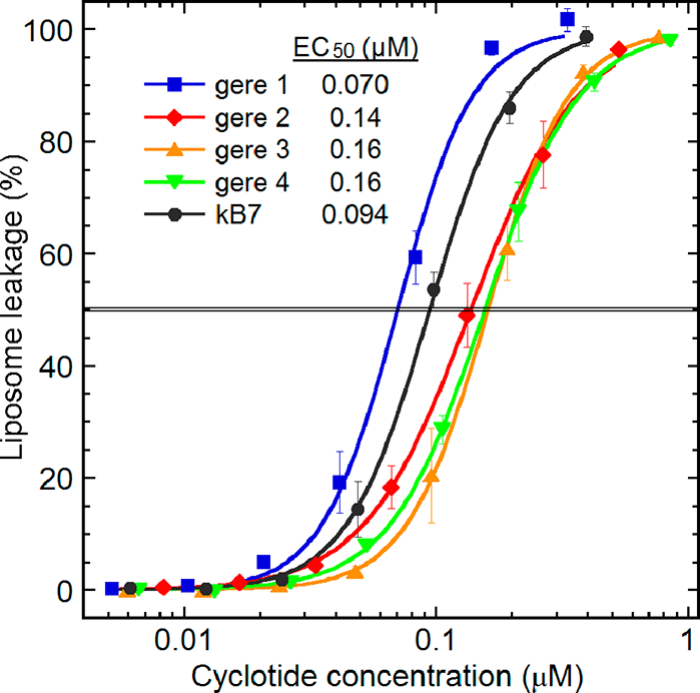
Cyclotide-induced membrane permeabilization assayed using liposomes made from an *E. coli* polar lipid extract, quantified by the release of carboxyfluorescein at 45 min of cyclotide incubation. Each marker represents the mean leakage in Tris buffer, pH 7.4, with standard deviations from three experiments done at individual peptide concentrations, i.e., with no cumulative additions. The gere cyclotides exhibit typical cyclotide membrane-disrupting activity profiles with the cyO2-like gere 1 being the most potent, as highlighted by the indicated EC_50_ values. Previous liposome leakage studies on cyclotides report an EC_50_ of 0.091 μM for neutral cyclotides compared to 0.094 μM for kB7 in the current study and an EC_50_ of 0.076 μM for cyO2 compared to 0.070 μM for gere 1 in the current study.^43^

Vesicles made from *E. coli* polar lipid extract represent a generic bacterial model membrane in terms of physicochemical properties of the lipid composition. Cyclotides are membrane disruptive,^43,47,48^ with the exception of the two *Momordica* cyclotides MCoTI-I and -II that seem virtually membrane-inert. The ability of cyclotides to bind to and permeabilize membranes is not fueled by electrostatic and hydrophobic interactions alone but also by an affinity for phosphatidylethanolamine phospholipid (PE) that is unique to cyclotides. However, it appears that although PE typically constitutes a major part of bacterial phospholipids, disruption of PE-containing liposomes can only partially be connected with bacteriolytic activity.^43^ Notably, with the exception of the cycloviolacins, cyclotides stand out among AMPs in that liposome leakage activity on bacterial model membranes does not correlate well with their reciprocal antibacterial activity on, for example, *E. coli* bacteria in the laboratory conditions used so far. The liposome leakage resulting from the gere cyclotides (Figure 6) corresponded well with previous results on cyclotides, in that the cyO2-like gere 1 was the most membrane disruptive and was likewise confirmed as antibacterial. The other gere cyclotides are less liposome disruptive but are still at a level for a typical AMP, suggesting strong antibacterial properties. Yet these cyclotides failed to exhibit any corresponding antibacterial activity against *E. coli* bacteria within the concentration range used in the present study, similar to other cyclotides with a neutral or negative net charge studied previously.^43^ The liposome leakage activity follows the cyclotide electrostatic properties with gere 1 (net charge of two) followed by kB7 (net charge one) and then the charge-neutral gere cyclotides. Since the liposomes, as do their bacterial counterparts, have a strong negative surface charge, it is reasonable to expect electrostatic parameters to dictate activity more than hydrophobic properties.

### Cytotoxicity Activity Results

**Figure 7:**
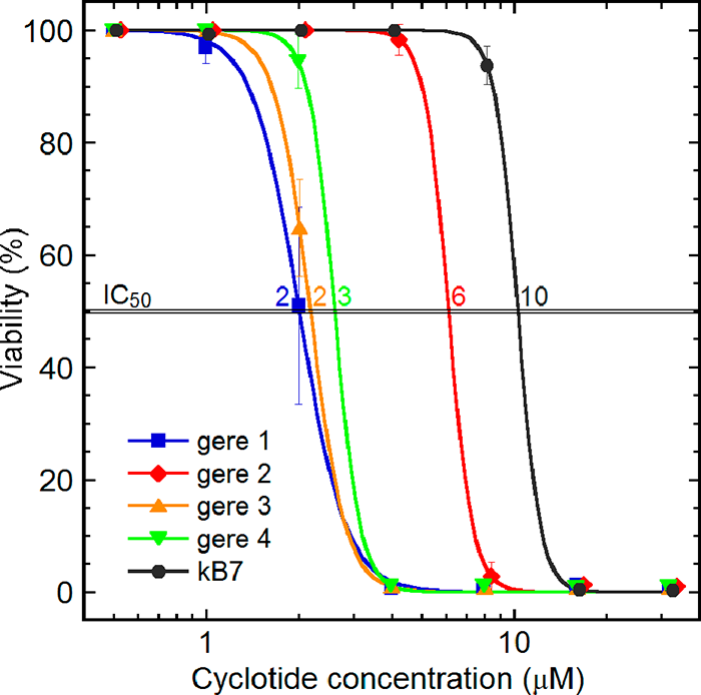
Cyclotide cytotoxicity using the U-937 human lymphoma cell line, measured as cell viability as a function of peptide concentration. This fluorometric microculture cytotoxicity assay quantifies viability by hydrolysis of the probe fluorescein diacetate by esterases in cells with intact plasma membranes. The cyclotides gave an overall toxicity range from 100% to 0% viability spans, at 0.1–15 μM, with the IC_50_ values indicated.

The cytotoxicity of the cyclotides was determined using the fluorometric microculture cytotoxicity assay, employing the U-937 a human lymphoma cell line. This assay is based on hydrolysis of the probe, fluorescein diacetate (FDA), by esterase in live cells containing an intact plasma membrane. The cyclotides were tested at a series of concentrations obtained by 2-fold dilutions. All cyclotides showed potent cytotoxicity (Table 2). The gere 1 showed the highest activity with an IC_50_ value of 2.0 μM and kB7 showed the least activity (IC_50_ 10.2 μM). The other three cyclotides showed activity in the range between the gere 1 and kB7 cyclotides. The survival index percentage of cytotoxic cells against the tested concentrations of each cyclotide is shown in Figure 7, where almost all cells were dead above a concentration of 20 μM.

For the new *Geophila* bracelet cyclotides, the net charge appears to have a more prominent effect on cytotoxicity than hydrophobicity, because gere 1 was the most potent cytotoxic cyclotide of all, most likely as a result of the influence of the +2 positive charge. As with previously reported cyclotides, presumably the positively charged residues in loops 5 and 6 together with a conserved Glu in loop 1 form a bioactive patch in gere cyclotides that modulates membrane binding.^29,47^ The resulting high affinity for negatively charged cell membranes, or the polar head groups of the phospholipid membranes, most likely underpins the high cytotoxic potency of gere cyclotides. Human cells and, in particular, cancer cell lines still have a distinct negative charge on their cell membranes, despite having a much weaker negative surface charge compared to bacteria. Although hydrophobic interaction is comparatively more important in human cell membrane disruption, electrostatics continues to be influential to a substantial extent. A similar tendency is seen for cyclotides in general and cycloviolacins in particular.^43^ For example, the higher cytotoxicity of *H. enneaspermus* cyclotides, hyen D compared to hyen E,^29^ and the *V. biflora* cyclotides, vibi G compared to vibi E, is proposed as being influenced by similar charge interaction.^49^

Hydrophobicity can be a difficult factor to predict from primary sequences and retention times, as its effect on activity on a lipid bilayer is influenced by the clustering and directions of hydrophobic surface areas.^50^ The distribution of hydrophobic residues forming a large patch at the surface of the molecule is less dispersed in bracelet cyclotides than Möbius cyclotides. While the bioactive patch is shown to be important for the recognition of PE groups, the hydrophobic patch is important for membrane insertion. By investigating the structure–activity relationships of cyO2 via chemically synthesized mutants, it has been shown that cyO2 binds membranes via loops 2 and 3, which is a different binding orientation from Möbius cyclotides.^51^ In the case of geres 2 and 3, with both having a neutral net charge, gere 3 with a high hydropathy index was more cytotoxic than gere 2 with a comparatively lower value. Most likely the structural differences in loop 2, loop 3, and loop 6 regions as shown in Figure 4 likely influence the nature of the hydrophobic patch of gere 2, which in turn affects its membrane insertion ability. Here, it appears that the influence of hydrophobicity comes into play when the net charge of the peptides is neutral. A similar trend was followed for kB7, the Möbius member with the lowest cytotoxicity in the FMCA and notably having the lowest hydropathy index of all cyclotides tested. Although kB7 has a +1 positive charge, the influence of charge interaction with the membranes is presumably not strong enough to compensate for the loss in hydrophobicity, leading to a very low overall cytotoxicity.

### Cyclotides Are Found in the Epidermis Region and Tissues of the Vascular Bundles

**Figure 8:**
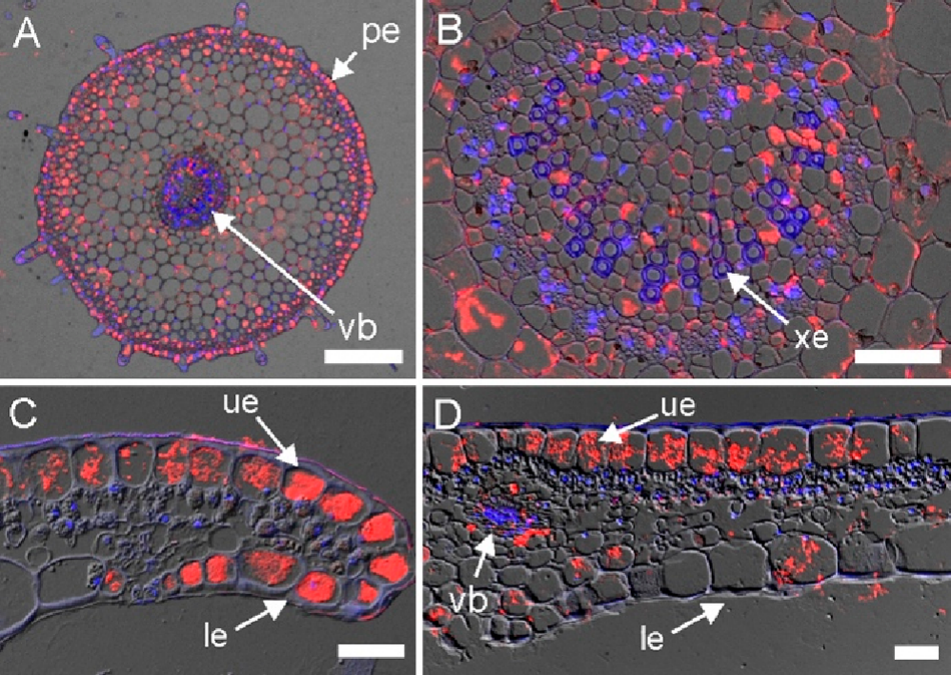
Immunohistochemical detection of cyclotides in *G. repens* petiole and leaf cross-sections. (A) Petiole section with cyclotides accumulating in the epidermis region (pe) and in the vascular bundle (vb). (B) Close-up on the petiole vascular bundles with xylem vessels (xe) devoid of cyclotides. (C) Cross-section of a leaf tip showing large quantities of the peptides in the upper (abaxial, ue) and lower (adaxial, le) epidermis. (D) Leaf blade cross-section with cyclotides in the upper epidermis and in tissues surrounding the leaf vein vascular bundle. DIC (Nomarsky contrast), DAPI, and DyLight 550 channels are merged, and the locations of cyclotides and nuclei are revealed by red and blue fluorescence, respectively. Bar = 200 μm (A); 50 μm (B, C, D).

To decipher the distribution of a group of cyclotides, similar to cyO2 in *G. repens* tissues, the immunohistochemistry techniques were applied. The detection of the fluorescence signal, specific for the fluorescence dye used, indicated that the antibodies raised against cyO2 bind to cyclotides present in *G. repens* (Figure 8). High cyclotide concentrations were detected in the lower and upper epidermis regions of the leaves and petioles, in comparison to other tissues assessed (the leaves and roots) (Figure 8). In the leaf blades, the majority of cyclotide material is accumulated in the upper (abaxial) epidermis and in cells surrounding the leaf vein vascular bundle (Figure 8D). The antibodies utilized herein were proven previously to be specific to cyclotides similar to cyO2.^52^ Therefore, confidence can be expressed that the immunohistochemistry techniques used herein allowed the depiction of the distribution of at least one cyclotide, namely, gere 1, which is different from cyO2 only at two positions.

The distribution of these peptides in *G. repens* was very similar to that observed in *Viola odorata*.^10,52^ In both species, belonging to different plant families, the peptides were present in the highest abundance in the epidermis and vascular tissues. In previous studies, such a distribution has been linked to biological roles in plant host defense against pathogens or sucking insects.^53^ It appears that the two nonrelated species have evolved independently very similar peptides together with their specific distribution in tissues and organs. This can be considered a strong example of the evolutionary convergence of cyclotides and their biological roles.

Although it has been known for some time that kB7 targets the human oxytocin/vasopressin signaling system, the role of kB7 as a partial agonist of insect inotocin receptors (homologous receptors to the oxytocin/vasopression system of vertebrates) was only reported recently.^54^ Despite a limited understanding on how cyclotides interact with insects, the latter finding nevertheless provides strong support for cyclotide herbivore defense that could be mediated via inotocin-like receptors. In this context, the observation that kB7 occurs in two different hosts, *O. affinis* and now *G. repens*, is supportive of its functional relevance to the host plant. It is postulated that inotosin receptors are of physiological importance in terms of egg-laying behavior, vascular and gut contractility, and for the learning behavior of insects. Although, the involvement of cyclotides in cell signaling or interaction with a target receptor has not been shown experimentally, the latter study points convincingly toward the involvement of cyclotides in host defense.

Cyclotides have been found in all Violaceae plants screened to date, but their presence is limited to only certain Rubiaceae plants. In the current study, cyclotides were found only in the *G. repens* of all the Rubiaceae plants screened. As Gruber and his colleagues point out, if cyclotides are inherited by a common ancestor following the principles of divergent evolution, it is unlikely that the cyclotide genes get lost in a significant portion of some plant families and not others.^20^ The observed distribution of cyclotides is more supportive of the convergent evolution of cyclotides, where cyclotide-bearing plants have evolved independently the ability to produce cyclotides in distant families. Supportive evidence for the convergent evolution proposal was obtained in this study by the discovery of gere 1 and gere 2, which are very similar to cyO2 and hyfl J, respectively, from a distant plant lineage (Violaceae). Most likely the ancestral cystine knot containing linear peptides, upon obtaining the feature of a C-terminal conserved Asn, have acquired the ability to recruit existing AEPs for cyclization. Some *Geophila* AEPs and PDIs show a more sequence homology to counterpart enzymes from the phylogenetically distant Violaceae family than to enzymes from the closely related *Oldenlandia* genus. At this point, the significance of the latter finding is not fully understood, but it is likely that cyclotide precursors together with their biosynthetic enzymes, driven by the need for host defense, have evolved independently in different plant families.

The novel gere cyclotide sequences reported herein enhance the understanding on how amino acid charge interaction and hydrophobicity may affect the cytotoxicity of cyclotides. This also gives a valuable opportunity to modulate cyclotide properties to increase their cell electivity toward the killing of cancer cells and the reduction of nonspecific toxicity. So far, structure–activity studies of bracelet cyclotides have been primarily limited to Möbius cyclotides, due to the absence of a robust method of synthesis and in vitro folding. With new studies tackling the folding problem of bracelet cyclotides, such structure–activity studies are becoming more feasible.^55^ Most recently, the potential of molecular grafting of the cyclotide scaffold for the treatment of bacteremia, a life-threatening condition with viable bacteria in the blood, was highlighted successfully.^14^ Grafted cyclotides showed broad-spectrum antibacterial activity against several multi-drug-resistant pathogens and clinical isolates from patients suffering from cystic fibrosis. The most active antibacterial cyclotide was found to be extremely stable in serum, with a low toxicity to erythrocytes, and provided protection in vivo in a murine model of *P. aeruginosa* peritonitis. In light of these recent findings, the *Geophila* cyclotides reported including gere 1 that exhibit potent antibacterial activity seem worthy of further structure–activity and grafting applications. Another important area for future studies will be to investigate the mechanistic behavior of gere cyclotides at concentrations below their IC_50_ values. In a recent study involving similar cytotoxic cyclotides, cell internalization without compromising membranes was demonstrated at sublethal concentrations.^29^ Thus, identifying new modes of action and molecular targets of gere cyclotides at sublethal concentrations is recommended to advance new therapeutic avenues for gere cyclotides.

In conclusion, of 50 plants screened from the Fabaceae, Rubiaceae, and Solanaceae families, *G. repens* from the Rubiaceae obtained in Sri Lanka was determined to contain cyclotides, leading to the discovery of a series of new bracelet cyclotides. These bracelet cyclotide scaffolds provide a unique opportunity to explore further structure–activity relationships in terms of antibacterial activity and cytotoxicity. Among the new cyclotides, gere 1, comprising a sequence very similar to cyO2 and the previously characterized kB7 cyclotide, were identified. Additionally, transcripts of four new AEPs and two PDIs likely involved in cyclotide biosynthesis were identified. Gere 1 was expressed in the transcriptome and at peptide level in *G. repens* albeit at different geographical locations, indicating that it likely serves a key function and its significance to the plant. In all assessed tissues, namely, the leaves, petioles, and roots, immunohistochemical analysis showed the epidermal distribution of cyclotides. Large cyclotide concentrations were detected in the epidermis of the leaves, especially in the lower and upper epidermis. All cyclotides displayed cytotoxic effects, which together with their membrane permeabilizing activity and tissue localization in the plant provide strong support that they play a crucial role in protecting *G. repens*.

## Experimental Section

### General Experimental Procedures

Preparative RP-HPLC and analytical RP-HPLC were carried out using a Shimadzu LC 10 system equipped with a photodiode array detector operating at 215, 254, and 280 nm wavelengths. Fractions from preparative RP-HPLC were collected at 8 mL/min for 1 h, yielding 60 fractions using a gradient of 5–60% solvent B (solvent A: 0.1% formic acid in 5% CH_3_CN and solvent B: 0.1 formic acid in 100% CH_3_CN). Individual cyclotides were further purified by analytical RP-HPLC with a flow rate of 1 mL/min on a Phenomenex column (C_18_, 250 × 4.6 mm, 5 μm) using a gradient from 15% to 55% of solvent B. Fractions were analyzed using MS, and their purity was further assessed as >95% by UV spectroscopy (215 nm). The pure cyclotides and fractions containing cyclotides (0.1 mg/mL) were analyzed on a nano Acquity UPLC-QTOF mass spectrometer (Waters, Milford, MA, USA) for the mass range of 300–2000 Da using a nano LC Waters C_18_ column (150 × 1.0 mm column, 5 μm) with a 0.30 μL/min flow rate for 70 min. Each mass spectrum was obtained in the positive-ion mode, and the obtained data were processed using MassLynx V4.1 software. Freeze-dried peptides (0.3–1 mM) were dissolved in 220 μL of H_2_O–D_2_O (9:1, v/v) at pH 4.5, and one- and two-dimensional NMR spectra (^1^H TOCSY and ^1^H NOESY) were recorded at 298 K on a Bruker Avance 600 MHz spectrometer equipped with a three-channel cryo probe (TCI: RPHe TR-^1^H and ^19^F/^13^C/^15^N 5 mm-EZ). All data, including TOCSY (mixing time 80 ms) and NOESY (mixing time 200 ms), were recorded and processed using Topspin (Bruker). Generally, 4096 data points were collected in the F2 dimension and 256 (128 complex) points in F1, with 512 increments of 8 scans over 11 194 Hz.

### Plant Material

Whole plants (roots, leaves, stem) of 50 plants were collected based on their traditional Ayurvedic usage (Table S1, Supporting Information).^22^ The plant specimens were identified at the National Herbarium at the Royal Botanical Garden, Peradeniya, Sri Lanka, by N. P. T. Gunawardena. A voucher specimen (no. UOC/NPSR/010) of *G. repens* and the other plants studied for cyclotide screening were deposited in the herbarium of the Department of Plant Sciences, University of Colombo, Sri Lanka. *G. repens* was collected from Bandaranayake Memorial Ayurvedic Research Institute, Mahargama (6°51′21″ N 79°54′56″ E) for large-scale extraction and immunohistochemistry assessment. The material for all RNA-seq analyses was obtained from *G. repens* plants cultivated at Uppsala University Botanical Garden (1987-3130*A; collected by Lars Jonsson on March 11, 1987, Cook Islands; collection no. 2506B).

### Small-Scale Extraction for Cyclotide Screening

Fifteen species from the Rubiaceae, seven from the Solanaceae, and 28 from the Fabaceae were collected according to their local Ayurvedic uses and possibility of the presence of cyclotides. Initially, ∼5–10 g of the air-dried aerial part of plant material was powered using a grinder and extracted with methanol (CH_3_OH) and dichloromethane (CH_2_Cl_2_) (1:1 v/v, 100 mL). Crude extracts were obtained by evaporating the solvent under reduced pressure at 40 °C using a rotary evaporator (Buchii, model-R-200). Crude extracts were stored under 4 °C until use. Crude extracts were redissolved in a mixture of CH_3_OH–CH_2_Cl_2_ (1:1 v/v, 25 mL) and solvent–solvent partitioned with Milli Q water (15 mL) to obtain the aqueous fraction. Aqueous extracts were freeze-dried and dissolved in 10% acetonitrile (CH_3_CN) for the identification of cyclotide-like masses (2–4 kDa) using LC-MS.

### Large-Scale Extraction and Isolation of Cyclotides

The powdered, air-dried whole plant (leaves, stem, and root) of *G. repens* (140 g) was extracted using 60% aqueous CH_3_OH (1.4 L). The mixture was filtered, and hydrophobic constituents were removed by liquid–liquid extraction with CH_2_Cl_2_ (2 × 700 mL). The aqueous fraction was rotary evaporated to remove CH_3_OH and freeze-dried to obtain 21.42 g (15.3%) of crude extract. The following ion-exchange protocol was used to concentrate the positively charged cyclotides. Ion-exchange material in a Buchner funnel (about 100 g) was activated with 100% CH_3_OH and then equilibrated with 10% aqueous CH_3_CN and 0.05% trifluoroacetic acid (about 0.5–1 L). The aqueous crude extract (10 g) was redissolved in 10% CH_3_CN and mixed with the ion-exchange material in a large beaker by stirring for 10 min. The material was then filtered through a Buchner funnel fitted with a filter paper (Whatman, 110 mm, pore size 11 μm). Ion-exchange material was washed with 10% CH_3_CN containing 0.05% trifluoroacetic acid (0.5 L). Bound cyclotides were eluted with 0.5 M NaCl in 10% CH_3_CN containing 0.05 trifluoroacetic acid (1 L) and loaded onto a reversed-phase preparative HPLC.

### Reduction, Alkylation, and Enzymatic Cleavage of the Cyclotides

Reduction of the disulfides, alkylation of the cysteines, and enzymatic cleavage of the linearized cyclotide backbone were carried out for sequence analysis. Peptides were reduced with dithioerythriol in 0.25 M Tris-HCl (pH 8.5) containing 4 mM EDTA and 8 M guanidine-HCl and incubated at 37 °C in the dark and under N_2_ for 3 h. Iodoacetamide (50 mg, in 0.5 M Tris-HCl, 2 mM EDTA) was added to alkylate-free thiols. The alkylation reaction was quenched after 10 min by adding 250 μL of 0.5 M citric acid. The reduced and alkylated peptides were purified by size-exclusion chromatography (Sephadex G-25 based PD 10 column, Cytiva) and cleaved by incubation with either trypsin or endoprotenase GluC enzyme dissolved in 50 mM NH_4_HCO_3_ buffer (pH 7.8) at 37 °C, overnight. The MS/MS fragmentation of peptides resulting from enzymatic cleavage was determined using UPLC-QToF nanospray MS (Waters nanoAcquity, 75 μm × 250 mm 1.7 μm BEH130 C_18_ column and/or Waters QToF Xevo). The MS/MS fragmentation spectra were processed using MaxEnt 3, and the b- and y-ions were assigned in the Peptide Sequencing module in the MassLynx software (Waters).

### Transcriptome Mining for Cyclotides

*G. repens* plant material (leaves) was frozen in liquid nitrogen and stored at −80 °C until RNA isolation. Frozen tissue (∼100 mg) samples were powdered in liquid nitrogen using a chilled mortar with a pestle, and, next, total RNA was extracted according to the manufacturer’s guidelines (NucleoSpin RNA Plant, Macherey-Nagel GmbH & Co. KG, Düren, Germany). The RNA-seq and transcriptome mining for cyclotide sequences was performed utilizing previously established protocols and methods.^33^ The RNA sample was shipped to the external sequencing service provider (Macrogen Europe B.V. Meibergdreef 57 1105 BA, Amsterdam, The Netherlands) in RNase-free water on dry ice. The cDNA library was prepared using a TruSeq stranded mRNA kit (Illumina, San Diego, CA, USA), followed by Illumina Novaseq 2 × 150 bp paired-end sequencing (total of 100 mln reads). The transcriptome was de novo assembled using Trinity^56^ and mined for sequences similar to the cyclotides from Cybase (retrieved 19.06.2021; http://www.cybase.org.au/) using NCBI-BLAST+ and a motif search (C-x(0,1)-[ES]-S-C-[AV]-[MFYW]-I-[PS]-x(0,1)-C) performed using Fuzzpro of EMBOSS (v. 5.0.0). Sequences containing six conserved cysteines in positions aligning with previously known cyclotides and aspartic acid (D) or asparagine (N) at the C-terminal were considered cyclotides. The acyclotides are the absence of D/N at the C-terminal or absence of a stop codon in the sequence.

### Determination of Minimum Inhibitory Concentration

The dried cyclotides were subjected to MIC determination^43^ against *Staphylococcus aureus* (ATCC 29213), *Escherichia coli* (ATCC 25922), *Pseudomonas aeruginosa* (ATCC 27853), and *Candida albicans* (ATCC 90028). The cyclotides (stock concentration of 65 μM) were dissolved in Tris buffer for the assay, and a concentration gradient of the cyclotides was screened against a microbial suspension mixed in a 1:1 ratio in each well (50 μL:50 μL). Microorganisms were grown in 3% tryptic soy broth (TSB) at 37 °C overnight, after centrifuging at 2000 rpm for 6–7 min, and bacterial pellets were washed twice and dissolved with Tris buffer. The absorbance was measured at 600 nm, and the solution was diluted to a concentration of 1 × 10^6^ bacteria/mL. Dilution series were prepared using a serial dilution method with cyclotides and Tris buffer. In each plate, 50 μL of microbial suspension (approximately 50 000 cells/well) was added and incubated for 5 h. After incubation, 5 μL of 20% TSB was added to each well. Each plate was incubated based on the growth cycle of the microbe for 6, 9, 10, and 12 h at 37 °C. The minimum concentration that inhibited 100% growth was recorded as the MIC value. Triplicate assays were carried out for each sample.

### Liposome Assay

Liposomes were manufactured and their permeabilization was assayed as described previously.^48^ Briefly, by dissolving an *E. coli* polar lipid extract in chloroform and evaporating the solvent under agitation, with a nitrogen gas flow, and then storing in vacuum, dry films were formed on the walls of a glass flask. Lipid films were resuspended in an aqueous solution of 100 mM 5(6)-carboxyfluorescein in 10 mM Tris (set to pH 7.4 at 37 °C). Suspensions were subjected to repeated extrusion through a 100 nm polycarbonate membrane in order to reduce multilamellar structures and polydispersity. Untrapped carboxyfluorescein was removed by gel filtration. Membrane permeability was measured by monitoring carboxyfluorescein efflux from the liposomes to the external low-concentration environment, resulting in loss of self-quenching and an increased fluorescence signal. The 96-well plates were prepared with a 2-fold serial dilution of the peptides in Tris buffer, as well as controls without peptides (background) and 0.16% Triton X-100 (maximum leakage). The plates were preheated to an incubation temperature (37 °C) and administered a liposome suspension, to a final lipid concentration of 10 μM in 200 μL. The effects of each peptide concentration on the liposomes were monitored for 45 min, at which point the initial leakage had largely subsided. The results obtained represent the mean from triplicate experiments with standard deviations and are expressed as percent of total leakage generated with Triton X-100 and subtraction of the baseline value. The EC_50_ values are calculated from a sigmoidal dose–response curve with a variable slope to the leakage percentage as a function of the peptide concentration (log 10).

### Cytotoxicity Assay

Cytotoxicity activities of the pure cyclotides were evaluated using a fluorometric microculture cytotoxicity assay (FMCA),^57^ which is based on the monitoring of fluorescence arising from fluorescein that is produced as a result of fluorescein diacetate (FDA) hydrolysis by cells with intact cell membranes. Human lymphoma cells (U937) suspended in cell-growth medium were dispensed into the microtiter plate containing pure cyclotides. Each well was seeded with 200 μL of cell suspension, containing approximately 20 000 cells, to give a total volume of 200 μL/well. The plates were then incubated for 72 h at 37 °C in a 5% CO_2_ atmosphere. After an incubation period, the plates were centrifuged at 1000 rpm for 5 min at 37 °C, the medium was removed by aspiration, and the cells were washed with PBS (80.0 mg of NaCl; Himedia, USA, 2.0 mg of KCl (Sigma), 14.4 mg of Na_2_HPO_4_ (Sigma), and 2.4 mg of KH_2_PO_4_ (Sigma), in 10.0 mL of distilled water). FDA (10 mg of FDA dissolved in 1 mL of 100% DMSO) was added to preheated (37 °C) Q2-buffer (40 mL of 125 mM NaCl, 10 mL of 25 mM HEPES added up to 400 mL with MQ-H_2_O, pH 7.4). A portion from this stock solution (100 μL) was then added to each well. After 40 min of incubation at 37 °C the fluorescence was measured at 485 nm excitation and 538 nm emission. The fluorescence in each well is proportional to the number of living cells, and cytotoxicity activity of a fraction is inversely proportional to fluorescence intensity. The activity of the cyclotides was reported in terms of % viability, which was defined in terms of the fluorescence in the experimental wells, expressed as a percentage of that in the control wells after the fluorescence of the blanks had been subtracted from both the experimental and the control readings. The IC_50_ values were calculated from a sigmoidal dose–response curve with a variable slope to the percentage viability as a function of the peptide concentration (log 10).

### Immunohistochemical Analysis

The current study employed polyclonal anti-cyclotide antibodies raised for a previous study^52^ by Capra Science Antibodies AB, Angelholm, Sweden. The specificity of the antibodies was tested in a series of dot blot and Western blot experiments, and the antibodies bound to different cycloviolacin cyclotides with affinity varying depending on their sequence similarity to cyO2.^52^ The specimens were prepared and examined according to previously published protocols.^52^ Parts of *G. repens* (leaf blades, petioles) were fixed in 4% formaldehyde and 0.25% glutaraldehyde in microtubule-stabilizing buffer (MSB) composed of 50 mM PIPES (piperazine-*N*,*N*′-bis[2-ethanesulfonic acid]), 10 mM EGTA (ethylene glycol-bis[β-aminoethyl ether]-*N,N,N*′*,N*′-tetraacetic acid), and 1 mM MgCl_2_, pH 6.8. Afterward, the tissues were dehydrated in a graded ethanol series and embedded in Steedman’s wax, i.e., a 9:1 (w/w) mixture of polyethylene glycol 400 distearate and cetyl alcohol (Sigma-Aldrich). The 5 μm slides were mounted on the glass slides, rehydrated in graded ethanol/PBS series, and stained. Primary polyclonal rabbit anti-cyO2 antibody and secondary goat anti-rabbit Ig antibody conjugated with DyLight 549 (AS12 2084, Thermo Fisher Scientific) were used. The chromatin of the nuclei was stained with 7 μg/mL 40,60-diamidino-2-phenylindole dihydrochloride (DAPI, Sigma-Aldrich) in PBS. The stained sections were examined with a fully automated upright fluorescent microscope (Leica DM6000 B) equipped with a digital 5 megapixel color microscope camera with an active cooling system (Leica DFC450 C), a selection of lenses (HC PL FLUOTAR 109/0.30 dry, HCX PL FLUOTAR 409/0.75 dry, and HC PL APO 639/1, 40 oil), and an external light source for fluorescence excitation (Leica EL6000).

## Supporting Information Available

List of plants screened for the presence of cyclotides in this study, fragment ions of gere peptides in MS/MS, BLAST search in the *G. repens* transcriptome for AEP and PDI enzymes similar to enzymes from cyclic peptide bearing plants, cyclotide precursor sequences in the transcriptome (PDF)
