# Supplementary material for: Screening for Cyclotides in Sri Lankan Medicinal Plants: Discovery, Characterization, and Bioactivity Screening of Cyclotides from Geophila repens
Source: J Nat Prod. 2022 Dec 16;86(1):52–65. doi: 10.1021/acs.jnatprod.2c00674 (PMC9887600; doi:10.1021/acs.jnatprod.2c00674)
Supplement: Supplementary file 1 — np2c00674_si_001.pdf [file np2c00674_si_001.pdf]

Supporting Information

Screening for Cyclotides in Sri Lankan Medicinal Plants: Discovery, Characterization and Bioactivity Screening of Cyclotides from *Geophila repens*

Sanjeevan Rajendran,[†,‡] Blazej Slazak,[†,§] Supun Mohotti,[‡] Taj Muhammad,[†] Adam A. Strömstedt,[†] Małgorzata Kapusta,[∇] Emilia Wilmowicz,[‖] Ulf Göransson,[†] Chamari M. Hettiarachchi,[‡] Sunithi Gunasekera[*,†]

[†] *Phamacognosy, Department of Pharmaceutical Biosciences, Uppsala University, Biomedical Centre, SE 75124 Uppsala, Sweden*

[‡] *Department of Chemistry, Faculty of Science, University of Colombo, Thurstan Rd, Colombo 00300, Sri Lanka*

[§] *W. Szafer Institute of Botany of the Polish Academy of Sciences, 46 Lubicz, 31-512 Cracow, Poland*

[∇] *Department of Plant Cytology and Embryology, Faculty of Biology, University of Gdańsk, 59 Wita Stwosza, 80-308 Gdańsk, Poland*

[‖] *Faculty of Biological and Veterinary Sciences, Nicolaus Copernicus University, 1 Lwowska Street, 87-100 Toruń, Poland*

Email: [*]sunithi.gunasekera@farmbio.uu.se

Table S1. **List of Plants Screened for Presence of Cyclotides in this Study**

	Plant	Vernacular name	Voucher	Place of Collection	Presence of cyclotides
	**Family: Fabaceae**				
1	*Adenanthera pavonina* L.	Madatiya	UOC/NPSD /050	University of Colombo	NO
2	*Alysicarpus vaginalis* (L.) DC.	Aswenna	UOC/NPSD /051	Navinna ayurvedic garden	NO
3	*Bauhinia acuminata* L.	Ela koboleela	UOC/NPSD /052	Home garden, Kohuwala	NO
4	*Bauhinia purpurea* L.	Rath koboleela	UOC/NPSD /053	University of Colombo	NO
5	*Bauhinia racemose* Lam.	Nil kobolela	UOC/NPSD /054	University of Colombo	NO
6	*Butea monosperma* (Lam.) Taub.	Gas kela	UOC/NPSD /055	Navinna ayurvedic garden	NO
7	*Cassia alata* L.	Eth thora	UOC/NPSD /056	Home garden, Kohuwala	NO
8	*Cassia auriculata* L.	Ranawara	UOC/NPSD /057	Home garden, Kamburupitiya	NO
9	*Cassia fistula* L.	Ehela	UOC/NPSD /058	Home garden, Kamburupitiya	NO
10	*Cassia tora* L.	Pethi thora	UOC/NPSD /059	Home garden, Kamburupitiya	NO
11	*Crotalaria retusa* L.	Kaha andanahiriya	UOC/NPSD /060	Home garden, Kamburupitiya	NO
12	*Crotalaria verrucosa* L.	Nil andanahiriya	UOC/NPSD /061	Bellanwila Aththidiya mash Land	NO
13	*Derris Scandens* (Roxb.) Benth.	Kala wal	UOC/NPSD /062	Home garden, Mirigama	NO
14	*Desmodium heterophyllum* (Willd.) DC.	Udupiyaliya	UOC/NPSD /063	Home garden, Kamburupitiya	NO
15	*Erythrina variegata* L.	Erabadu	UOC/NPSD /064	Home garden, Kamburupitiya	NO
16	*Indigofera tinctoria* L.	Nil awariya	UOC/NPSD /065	Navinna ayurvedic garden	NO
17	*Mimosa pudica* L.	Nidi kumba	UOC/NPSD /066	Home garden, Kamburupitiya	NO
18	*Myroxylon balsamum* (L.) Harms	Kattakumanjal	UOC/NPSD /067	Navinna ayurvedic garden	NO

19	*Pterocarpus marsupium* Roxb.	Gammalu	UOC/NPSD /068	Navinna ayurvedic garden	NO
20	*Pterocarpus santalinus* L.f.	Rath hadun	UOC/NPSD /069	Navinna ayurvedic garden	NO
21	*Saraca asoca* (Roxb.) Willd.	Asoka	UOC/NPSD /070	Dathumalu Temple, Kohuwala	NO
22	*Sesbania grandiflora* (L.) Pers.	Kathuru murunga	UOC/NPSD /071	Home garden, Kamburupitiya	NO
23	*Tamarindus indica* L.	Siyabala	UOC/NPSD /072	Home garden, Kamburupitiya	NO
24	*Tephrosia purpurea* (L.) Pers.	Kathuru pila	UOC/NPSD /073	Navinna ayurvedic garden	NO
25	*Coccinia grandis* (L.) Voigt	Kowakka	UOC/NPSD /074	Home garden, Kamburupitiya	NO
26	*Luffa acyntangula* (L.) Roxb.	Dara watakolu	UOC/NPSD /075	Home garden, Kamburupitiya	NO
27	*Mukia maderspatana* (L.) M.Roem.	Heen kekeiri	UOC/NPSD /076	Home garden, Kamburupitiya	NO
28	*Trichosanthes cucumerina* L.	Pathola	UOC/NPSD /077	Home garden, Kamburupitiya	NO
	**Family: Rubiaceae**				
29	*Geophila repens* (L.) I.M.Johnst.	Koturu bedde	UOC/NPSR /010	Navinna ayurvedic garden	**YES**
30	*Guettarda speciose* L.	Nil Pichcha	UOC/NPSR /011	Pussellawa area, Central province	NO
31	*Haldina cordifolia* (Roxb.) Ridsdale	Kolon	UOC/NPSR /012	Navinna ayurvedic garden	NO
32	*Hedyotis auricularia* L.	Geta Kola	UOC/NPSR /013	Navinna ayurvedic garden	NO
33	*Knoxia zeylanica* L. **(endemic)***	*Ela ratmal*	UOC/NPSR /014	University of Colombo	NO
34	*Mitragyna parvifolia* (Roxb.) Korth.	Helamba	UOC/NPSR /015	Navinna ayurvedic garden	NO
35	*Morinda umbellata* L.	*Kiriwel*	UOC/NPSR /016	Navinna ayurvedic garden	NO
36	*Nauclea orientalis* (L.) L.	*Bakmi*	UOC/NPSR /017	Navinna ayurvedic garden	NO
37	*Oldenlandia biflora* L.	Pepiliya	UOC/NPSR /018	Navinna ayurvedic garden	NO

38	*Oldenlandia herbacea* (L.) Roxb.	Wal koththamalli	UOC/NPSR /019	Pussellawa area, Central province	NO
39	*Ophiorrhiza mungos* L.	Dathketiya	UOC/NPSR /020	Navinna ayurvedic garden	NO
40	*Paederia foetida* L.	*Apasu madu*	UOC/NPSR /021	Navinna ayurvedic garden	NO
41	*Pavetta lanceolata* Eckl.	Pavetta	UOC/NPSR /022	Pussellawa area, Central province	NO
42	*Spermacoce hispida* L.	Hin getakola	UOC/NPSR /023	Navinna ayurvedic garden	NO
43	*Wendlandia bicuspidate* Wight & Arn. **(endemic)***	Rawana idala	UOC/NPSR /024	Gelioya area, Central province	NO
	**Family: Solanaceae**				
44	*Datura metel* L.	Ela/Sudu attana	UOC/NPSR /025	Navinna ayurvedic garden	NO
45	*Solanum macrocarpon* L.	Wam batu	UOC/NPSR /026	Pussellawa area, Central province	NO
46	*Solanum melongena* L.	Ela batu	UOC/NPSR /027	Pussellawa area, Central province	NO
47	*Solanum nigrum* L.	Kalukanweriya	UOC/NPSR /028	Navinna ayurvedic garden	NO
48	*Solanum torvum* Sw.	Tibbatu	UOC/NPSR /029	Pussellawa area, Central province	NO
49	*Solanum virginianum* L.	Katuwel batu	UOC/NPSR /030	Pussellawa area, Central province	NO
50	*Withania somnifera* (L.) Dunal	Amukkara	UOC/NPSR /031	Navinna ayurvedic garden	NO

Table S2. **Fragment Ions Detected in MS/MS Fragmentation of Gere Peptides**

peptide	Enzyme	Fragments	Expected mass $(M+1)^+$	Observed mass fragments $(M+3H)^{3+}$ or $(M+2H)2^+$ or $(M+H)^+$
gere 1	Glu-C	SCVWIPCISSAIGCSCKNKVCYKNGIPCGE	3506.08	1169/ 1753.60/ 3506.57
	trypsin	NGIPCGESCVWIPCISSAIGCSCK	2713.13	2713.15
gere 2	Glu-C	SCAYFGCWIPGCSCKDKVCYINGIACGE	3333.80	1111.49/ 3332.47
	trypsin	VCYINGIACGESCAYFGCWIPGCSCK	3090.54	1030
	Glu-C + trypsin	VCYINGIACGE*	1255.43	629/ 1256.56
	Glu-C + trypsin	SCAYFGCWIPGCSCK	1853.13	927/ 1852.77
gere 3	Glu-C	SCVFIPCITTVIGCSCKDKVCTYNGVPCGE*	3483.03	1161.20/3482.54
	trypsin	VCTYNGVPCGESCVFIPCITTVIGCSCK	3239.77	1081.16
	Glu-C + trypsin	VCTYNGVPCGE*	1255.38	628.76/ 1256.50
	Glu-C + trypsin	SCVFIPCITTVIGCSCK	2002.20	1001.98/ 2002.91
gere 4	Glu-C	SCVFIPCFTTVVGCSCKDKVCYNNGVPCGE	3513.50	1171/ 3513.50

	trypsin	VCYNNGVPCGESCVFIPCFTTVVGCSCK	3272.76	1091
	Glu-C + trpsin	VCYNNGVPCGE	1268.38	635
	Glu-C + trypsin	SCVFIPCFTTVVGCSCK	2022.40	1011/ 2021.97
kalata B7	Glu-C	TCTLGTCYTQGCTCSWPICKRNGLPVCGE	3437.90	1146 / 3436.65
	trypsin	NGLPVCGETCTLGTCYTQGCTCSWPICK	3281.73	1094
	Glu-C + trypsin	NGLPVCGE*	844.37	846.40
	Glu-C + trypsin	TCTLGTCYTQGCTCSWPICK	2454.81	818 / 2454.11

*fragments with N to D deamination could be observed.

Table S3. **BLAST Search in the *Geophila repens* Transcriptome for AEP Enzymes similar to Verified Cyclizing Enzymes from Cyclic Peptide Bearing Plants**

	GrAEPs, Gene accession in transcriptome			
PALs, AEPs/GenBank accession	GrAEP1, DN162_c0_g1_i7*	GrAEP2, DN162_c0_g1_i4	GrAEP3, DN162_c0_g1_i10	GrAEP4, DN17346_c0_g1_i1
VyPAL1/QCW05334	306/467 (65.52%)	216/338 (63.91%)	300/463 (64.79%)	226/443 (51.02%)
VyPAL2/QCW05335	199/302 (65.89%)	198/302 (65.56%)	192/298 (64.43%)	152/276 (55.07%)
VyPAL3/QCW05336	271/409 (66.26%)	213/318 (66.98%)	254/405 (62.72%)	218/406 (53.69%)
VyPAL4/QCW05337	302/465 (64.95%)	216/338 (63.91%)	296/461 (64.21%)	225/443 (50.79%)
VyPAL5/QCW05338	184/274 (67.15%)	183/274 (66.79%)	176/270 (65.19%)	151/272 (55.51%)
VyAEP1/QCW05330	270/448 (60.27%)	201/318 (63.21%)	254/450 (56.44%)	321/490 (65.51%)
VyAEP2/QCW05331	273/448 (60.94%)	202/318 (63.52%)	253/432 (58.56%)	316/490 (64.49%)
VyAEP3/QCW05332	356/473 (75.26%)	261/344 (75.87%)	328/472 (69.49%)	272/445 (61.12%)
VyAEP4/QCW05333	357/474 (75.32%)	263/345 (76.23%)	328/473 (69.34%)	268/444 (60.36%)
HeAEP3/AWD84474	311/472 (65.89%)	222/344 (64.53%)	308/467 (65.95%)	242/475 (50.95%)
OaAEP1/KR259377	319/437 (73%)	246/340 (72.35%)	316/433 (72.98%)	238/439 (54.21%)
Butelase 1/KF918345	310/469 (66.1%)	220/341 (64.52%)	307/468 (65.6%)	244/449 (54.34%)
MCoAEP2/QFR54168	349/474 (73.63%)	252/347 (72.62%)	329/473 (69.56%)	262/443 (59.14%)
HaAEP1/6AZT_A	260/436 (59.63%)	207/324 (63.89%)	237/425 (55.76%)	307/485 (63.3%)
VbAEP1/QVD38651	252/385 (65.45%)	183/285 (64.21%)	245/381 (64.3%)	205/392 (52.3%)
VbAEP2/QVD38652	305/472 (64.62%)	223/344 (64.83%)	289/468 (61.75%)	235/489 (48.06%)
VbAEP3/QVD38653	352/473 (74.42%)	259/344 (75.29%)	323/472 (68.43%)	270/445 (60.67%)
VbAEP4/QVD38654	165/233 (70.82%)	166/233 (71.24%)	154/233 (66.09%)	180/274 (65.69%)

*DN162_c0_g1_i7: the accession DN162 indicates trinity read cluster, gene g1, and isoform i7. 357/474 (75.32%) indicates that 357 AA out of 474 AA in GrAEP1 match with VyAEP4. Enzyme hits with the highest sequence similarity are highlighted in yellow.

Table S4. BLAST Search in the *Geophila repens* Transcriptome for PDI Enzymes Similar to Verified Oxidative Folding Enzymes (PDI) from Cyclic Peptide Bearing Plants and Matching Percentage

	GrPDIs, Gene accession in transcriptome		
PDIs/GenBank accession	GrPDI 1, DN2699_c0_g1_i14	GrPDI 2, DN5047_c0_g1_i5	GrPDI 3, DN4143_c0_g1_i5
OaPDI/EF611425	443/537 (82.5%)	346/493 (70.18%)	157/471 (33.33%)
VbPDI 1/QVL22810	334/459 (72.77%)	337/458 (73.58%)	147/429 (34.27%)
GbPDI/GQ443304	35/96 (36.46%)	38/83 (45.78%)	61/97 (62.89%)
VbPDI 2/QVL22811	157/459 (34.2%)	160/444 (36.04%)	314/482 (65.15%)

Oa- *Oldenlandia affinis*

Vb- *Viola betonicifolia*

Gb- *Gloeospermum blakeanum*

Vy- *Viola yedoensis*

He- *Hybanthus enneaspermus*

Ha- *Helianthus annuus*

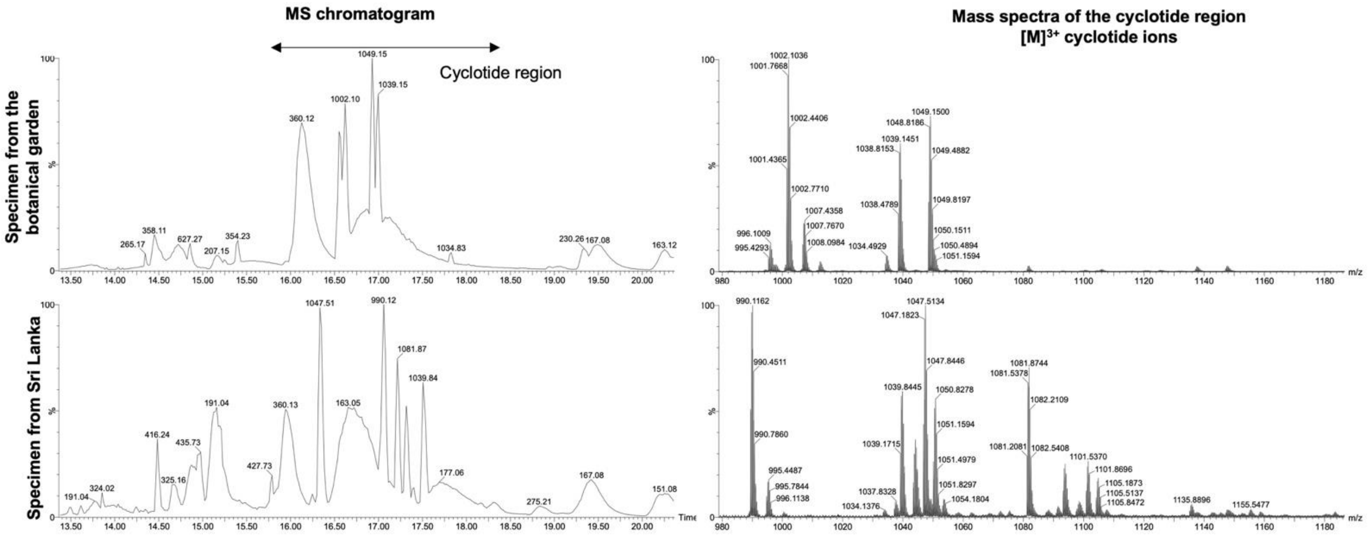

Figure S1. The comparison of the cyclotides found in the *G. repens* specimens originating from the Uppsala University botanical garden or Sri Lanka.

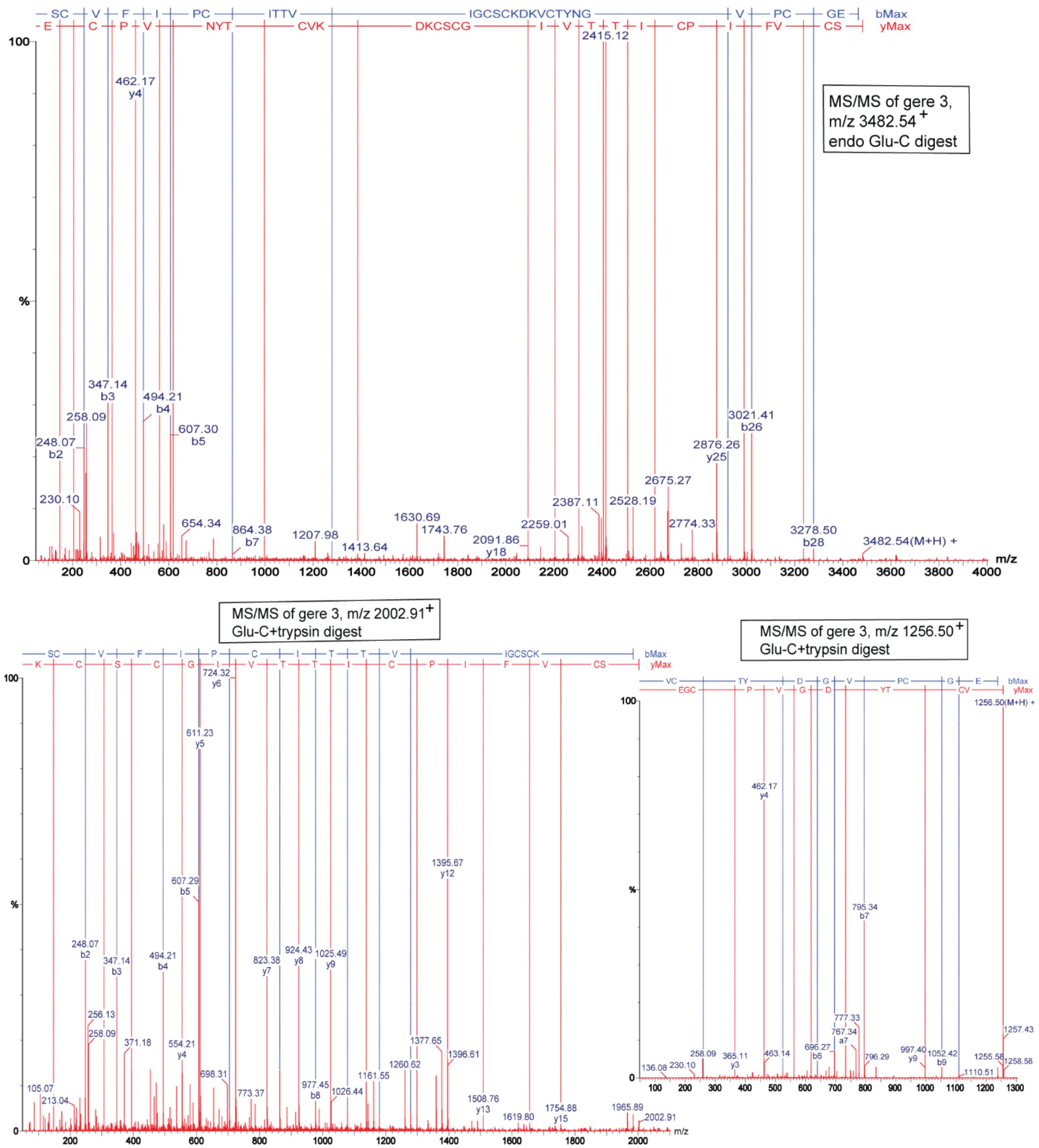

Figure S2. Gere 3 MS/MS fragmentation.

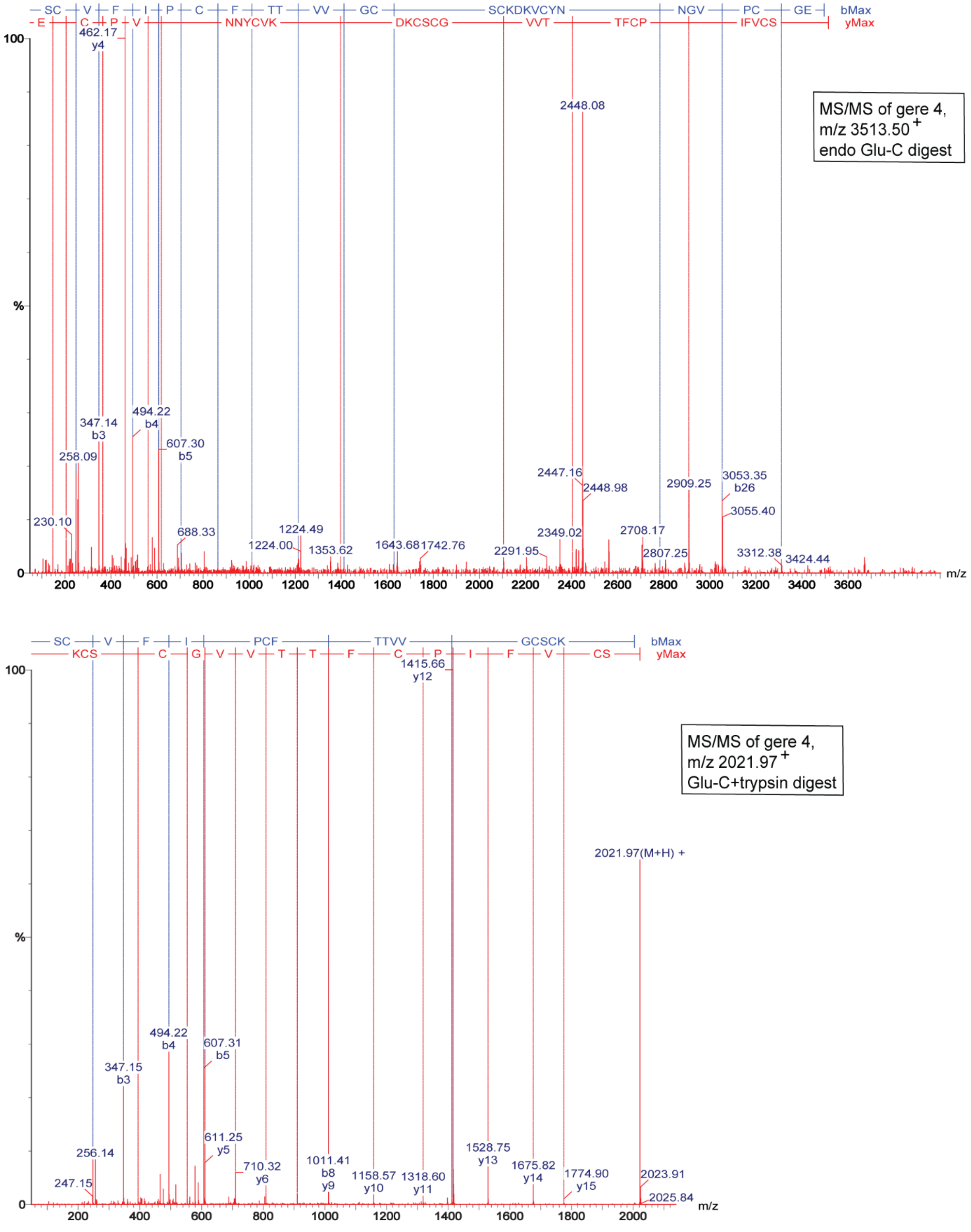

Figure S3. Gere 4 MS/MS fragmentation.

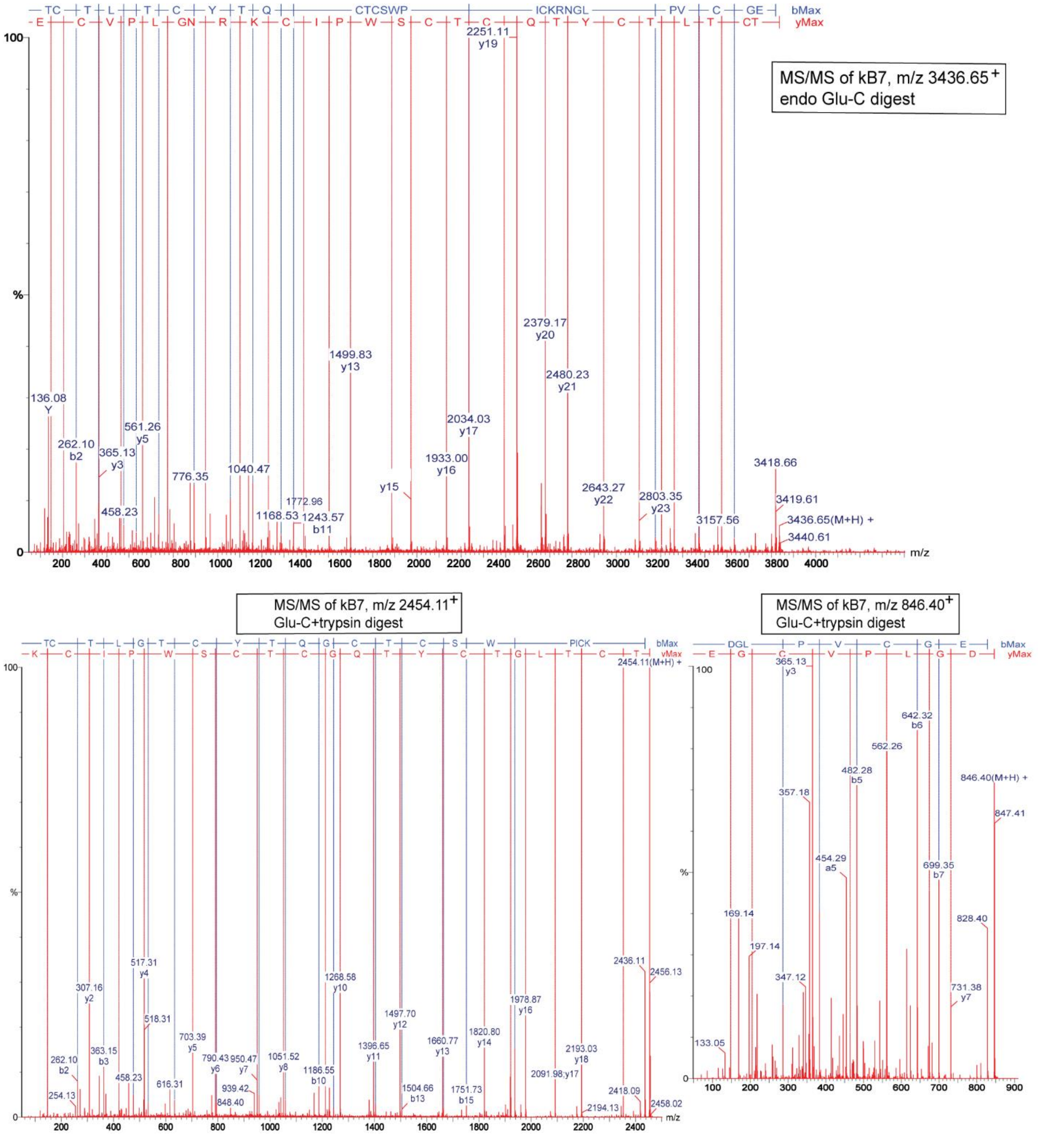

Figure S4. Kalata B7 MS/MS fragmentation.

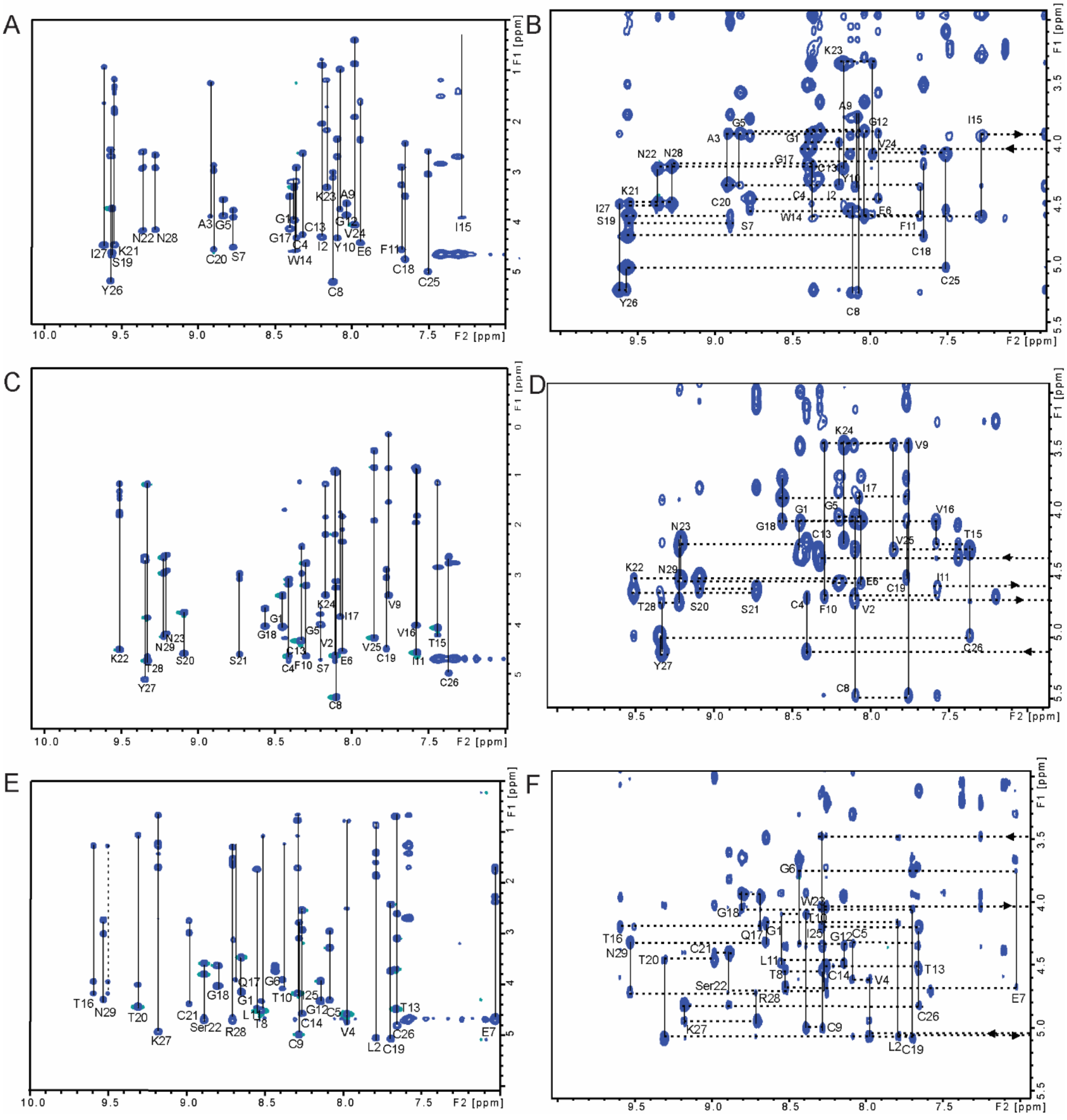

Figure S5. TOCSY and NOESY spectra of cyclotides isolated from *G. repens*.

Gere 3 (A, TOCSY and B, NOESY), gere 4 (C, TOCSY and D, NOESY) and kalata B7 (E, TOCSY and F, NOESY).

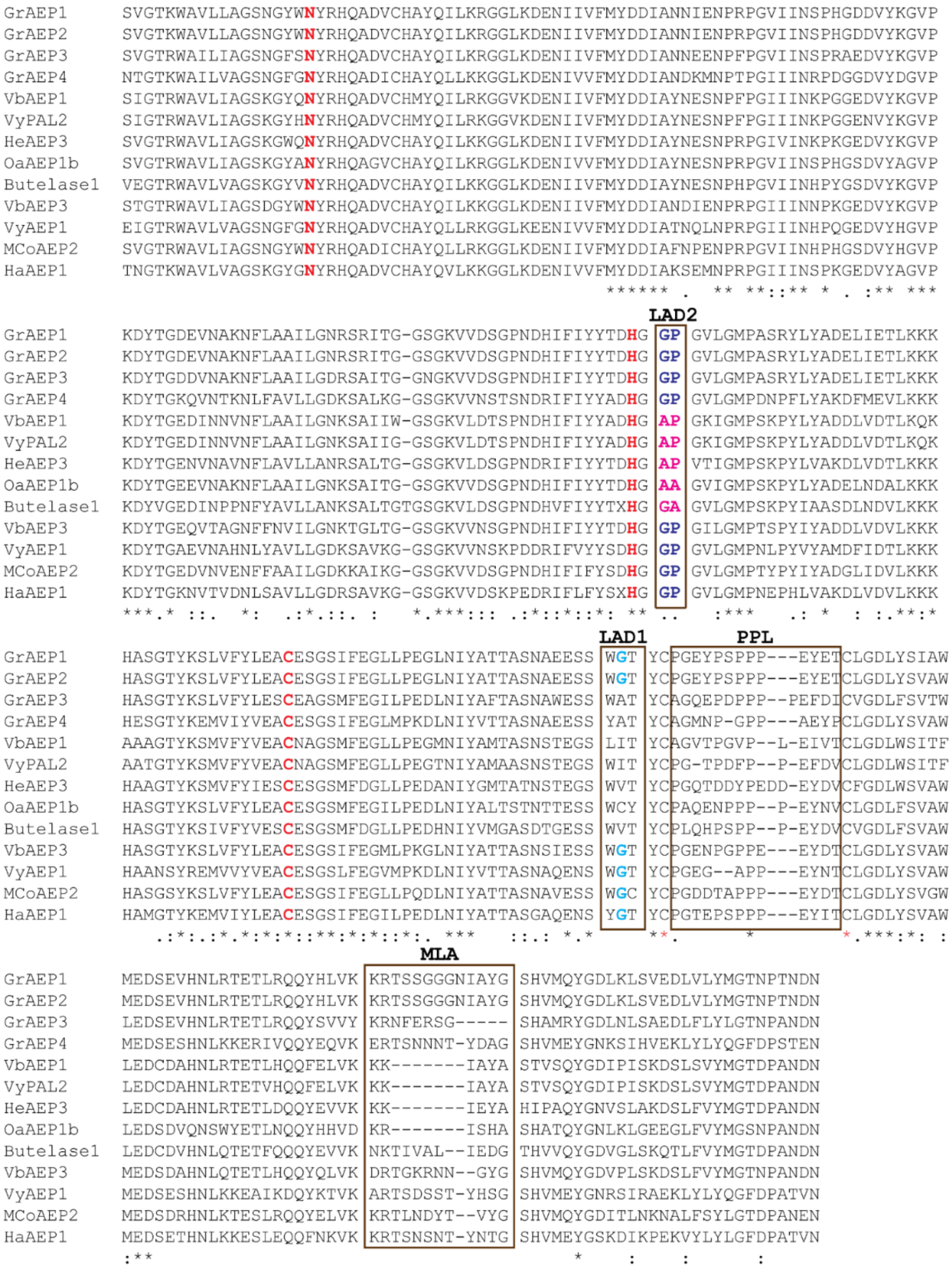

Figure S6. Multiple sequence alignment of *Geophila repens* AEPs (*Gr*AEP1-4) with functionally verified AEPs from cyclic peptide producing plants. The AEP conserved catalytic triad Asn, Cys and H are highlighted in red. Ligase activity determinant 1 (LAD1),[1] Ligase activity determinant 2,[1] poly-proline region[2] and marker of ligase regions[2] are highlighted. Following enzymes were used for the alignments: *Viola yedoendsis* VyAEP1[1], *Hybanthus enneaspermus* HeAEP3,[2] *Oldendlandia affinis Oa*AEP1b,[3] *Clitoria ternatea* butelase 1,[4] *Viola betonicifolia* AEP3,[5] *Momordica cochinchinensis* AEP2,[6] *Helianthus annus* *Ha*AEP1.[2]

```
GrPDI_1    YAPWCGHCQALEPIYNKLGKHLRGINSLVIAKMDGTTNEHPRAKAD--GFPTILFFPAGNKSFDPISFDGDRT
OaPDI      YAPWCGHCQMLEPTYNKLGKHLRGIDSLVIAKMDGTTNEHHRAKPD--GFPTILFFPAGNKSFDPIAFDGDRT
GrPDI_2    YAPWCGHCQALEPTYNKLAKHLRGIDSLVIAKMDGTTNEHPQARAE--GFPTLLFFPAGNKSSDPIPVETDRT
VbPDI_1    YAPWCGHCQSLEPTYNKLAKHLRDVDSLVIAKMDGTTNEHPRAKSD--GFPTLLFFPAGNKSFDPITVDTDRT
GrPDI_3    YAPWCGHCQKLAPVLDEVAVSFENDTDVVIAKFDGTANDFPQDIFDIQGFPTLYFKSASGKI---ELYDGGRS
GbPDI      YAPWCGHCKKLAPILDEVASSYQSDADVVIAKLDATANDYPTDTFEVQGYPTMYFRSASGNL---VQYDGDRT
VbPDI_2    YAPWCGHCKKLAPILDEVASSFLKEADVVIAKLDATTNDYPTDIFDVQGYPTLYFISSSGAL---VQYDGERT
           ********: * *  :::.       .:****:*.*:*:.     :  *:**: * .:..        :  *:

GrPDI_1    VKEFYKFLKKHSAIPFKLQKPATSSQTKPSDA-----NVSHDDSSSSEDLKDEL
OaPDI      VVELYKFLKKHATHPFKIQKPATSSP-QTKGS-----GVSQDESSTSKDLKDEL
GrPDI_2    VVALYKFLRKHASIPFKLQKPTSTPTSEDSDA------KEGSKSSTADVRKDEL
VbPDI_1    VVAFYKFLKKNAAIPFKLQKPASATKPETKPETSESKGSDEKSGSSSSEAKDEL
GrPDI_3    KEDLIDFIKNNS---------------EKV--------------TLQESRKDEL
GbPDI      KEAIIEFIEKNR---------------DKVAQ------------QEQEPAKDEL
VbPDI_2    KEHIIEFIENNR---------------NKAPE-----AVEQEAEAEAETAKDEL
              : .*:.::                .                   .  ****
```

Figure S7. Multiple sequence alignment of *G. repens* protein disulfide isomerases, *Gr*PDI1-3 with previously reported PDIs from Rubiaceae (*Oa*PDI)[7] and Violaceae (*Gb*PDI),[8] (VbPDI)[5] The active site residues CGHC are highlighted.

Data S1. Cyclotide precursor sequences identified in *Geophila repens* transcriptome. The mature cyclotide sequences are marked in grey in the translated protein sequences; the stop codon in the translated sequence is indicated by an asterisk (*).

> Scaffold cDNA sequences- cyclotide gere 1 - DN21349_c0_g3_i1

AAATGGGACTGAACAATAATTGATTGTGGAGACTGTATGCTTCGTCTCACACCTATAAAACC
TATACTTCAACCCACAGCAAACTCATCAAAACTAGTTCCGTGAGATTATTCCTTCAACGAAT
TCTGCAAAGTGGAATTAATGGCTAAGTTTGCCAATTACTTCTTGCTCTTTTTGCTCATTGCAT
CACTTGTGATGTTGGACGTTCACTCTTCCAGCAATATGCAAGTCCCCGATATGGGAAGAAGG
CTGGTGATGAATTCTGACCCTAATGGGATACCATGTGGTGAATCGTGCGTTTGGATCCCGTG
CATCTCTTCTGCTATTGGCTGCAGTTGCAAAAATAAAGTCTGCTATAAGAATGGATTAGATC
CAGAGAGTATTTAATGTGGTGGGCCGTGCGGGCGTGCGCTACTCTTTATAGTAGCTTTCATG
GGATCTCTCGCGTGTCAACAATAATTATCCCTACAATTGCTTTCAGGCAATATTGGGGTTTGC
GATGATGTAATGTGTGTTGAAGTAATTTCATTTTCATGCATGTTTTGGTTTGGTTTGGTTTGGT
TTGGTTTGTGATGTGAATTGTTCTTGAATTCAACAAGGGCAATATATATATAAATAAAATCA
TTTCCTTGTTTCTTTTAAAAAAAA

> Translational precursor sequence-cyclotide gere 1

MAKFANYFLLFLLIASLVMLDVHSSSNMQVPDMGRRLVMNSDPNGIPCGESCVWIPCISSAIGCS
CKNKVCYKNGLDPESI*

> Scaffold cDNA sequences- cyclotide gere 5- DN6031_c0_g1_i2

CGGAATGCCAATTATTGTCCTTGTCTGAACAATTAATGTTTGAGGGAGACTGCTTCGCCTCAC
ATTCCTATAAAACCCTTATTTAATCCACTGCAAACTCACCAAAGCAGATTCTATCCTTGAGC
TTATAGTTTATCAGCGAAGTCTACAACTTAAGTGGAATATGGCCAAGTTTGCTACTCACCTC
ATAGTCTTTGTGCTCGTTGCGTTTGTGATGTTGGAAGTTCACTCAGCTACTACTATGCAAGTC
TCCGATTTGGGAAGAAGGCTAGTGATGAATCCTAACGGCGTTGCATGTGGTGAATCGTGCGC
TTATTTCGGGTGTTGGATTCCGGGCTGCAGCTGCAAAGATAAAGTCTGCTATTTTAATGGACT
TGAGCCTGAGGCTATTTAATGTGGTGGACCGTGCGGGTCTGCACTACTCTTTCATGGGATCTC
GTGTGTCAACAATAAGTATCCCTGCAATTGTTTCCGCGGCAATGTTGGGGTTTGTTATGCTTG
AATGTGTATTTAAGTAATGTGTGTGTGTGCTTTCTTTTTCATGTCCAAATAATAATGGTTTGTT

ATGCTTGAATGTGTATTTAAGTAATGTGTGTGTGTGCTTTCTTTTTCATGTCCAAATAATAAT
GGTTTGTT

> Translational precursor sequence-cyclotide gere 5

MFEGDCFASHSYKTLILIHCKLTKADSILELIVYQRSLQLKWNMAKFATHLIVFVLVAFVMLEVH
SATTMQVSDLGRRLVMNPNGVACGESCAYFGCWIPGCSCKDKVCYFNGLEPEAI*

> Scaffold cDNA sequences- cyclotide gere 6- DN21349_c0_g1_i1

AAGCTACTAATCCCTGCAGCTGAGAGAGCTTATAATTCCTTCAACAAATTCTGCCAAGTGAT
CAAATGGCTAAGTTTGCTAATTACCTCATGCTCTTTCTGCTCGTTGCATCACTTGTGATGATG
GAAGTTCACTCTTCCAGCAGCATCGAAGTCCCCGATCTGGGAAGAAGGCTGCTGATGAATCC
TGACTCTAACGGCGTACCATGTGGTGAATCGTGCGTTTTTATCCCTTGCTTGACATCTGTTGT
TGGCTGCAGTTGCAAAGATAAAGTTTGCTATAATAACGCACTCTAAAGGGTGAGAGTATTTA
ACGTGATTCATGGAATTCATGAAATTCTGTGTGTCACTAATAATTATCCCTAAAACTGCTTCC
CAGGCAATATTGGGGTTTGCTATATATATGCTTGTAATGTAAGGTGTGCTAAAATAATTTCCT
GCTATGCGGGTTTAGTTTGGTTTGTGATGTGTGCTTTCGGTTTCATGTTCAAATAATATTGGA
TTGCCTTTTGTGAATTATCCCTCAATTCAACAAAGGGTAATGTATAAATAAATAAAATCTTCT
CTTGTTAAAAAAAA

> Translational precursor sequence-cyclotide gere 6

MAKFANYLMLFLLVASLVMMEVHSSSSIEVPDLGRRLLMNPDSNGVPCGESCVFIPCLTSVVGC
SCKDKVCYNNAL*

> Scaffold cDNA sequences- cyclotide Gere 7- DN9814_c0_g1_i1

ATTTCCTTGAGCTTATTCCTTCAGCTAACTCTGCAAAGTGGAATTAATGGCAAAGTTTGCTAA
ATACCTTTTGCTCTTTTTGTTCACTGCATCAATTGTGATGCTCGAAGTTCAGTCTTCCAACGTT
ATTATGCAAGACCCCGATTTGGTAAGAAAGCTGGCTATGAATCTTGACCCTAACGGCTTACC
AACATGTGGTGAAACGTGCTTTACGGGCAAATGCTATACTCCTGGCTGCACCTGCGGCGCAT
ATTTTGTCTGCCTTAAGAATGGACTAGATCCAGAGAGAGTTTAATGCGGGCTGCATAACTCT
TTCTGCTAGCTTTCATGGGATCTTGTGTGACAATAATAATTATCCCTACAATCGCTTTGGAAG
CAATATTGGGGTTTGCTGTGCCTGAAGTTGTGTTTAAGTAATTTCATTTCGTTTGGTTTGGTTT

GTGACGTGTGCTTTCCTTTTTATGTCCAAATAATATTGTAAATTATCCTTTAATATAAGGGTA
ATGTATAAATAAATAAAATCCTCTCTTTGCTTCTTTTACTCTCATGCCC

> Translational precursor sequence-cyclotide gere 7

MAKFAKYLLLFLFTASIV**M**LEVQSSNVI**M**QDPDLVRKLA**M**NLDPNGLPTCGETCFTGKCYTPGC
TCGAYFVCLKNGLDPERV*

> Scaffold cDNA sequences- cyclotide gere 8- DN21349_c0_g2_i1

TACAAATTCCTGCAGAGAGAGGTCAAAATTTCTTCAACAGATTCTACAAATTAAGTGATCTA
ATGGCTAAGTTTGCTAATTACCTCATGCTCTTTCTGCTCGTTGCATCACTTGTGATGATGGAA
GTTCACTCGTCTGACACCATCGAAGTCCCCGATTTGGGAAGAAGGCTGCTGATGAATCCTGA
CTCTAACGGCATGCCATGTGGTGAATCGTGCGTTTGGATCCCGTGTCTCAGTGCTATTGCGG
GCTGCAGTTGCAAAAATAAAGTCTGTTATAATAATGCACTGTAAAGTGAGAGTATTTAAGGT
GGTGAACCGTGCAGCCGTGCGTTAATCTTTCTGTGTGCCAATAATAATAGTATCCCTACTATT
GCTTTCCAAGTAATATTGGGGTTTGCTTGTAGTGTATGTATGGTGTGCTTAAGTAATTTCTTG
TTTATGCATGTGATTTGGTTTGGTTTGTGATGTGTGTGCTTTCCGTTTCATGTCCAAATAATGT
TG

> Translational precursor sequence-cyclotide gere 8

MAKFANYL**M**LFLLVASLV**MM**EVHSSDTIEVPDLGRRLL**M**NPDSNG**M**PCGESCVWIPCLSAIAG
CSCKNKVCYNNAL*

References:

(1) Hemu, X.; Sahili, A. El; Hu, S.; Wong, K.; Chen, Y.; Wong, Y. H.; Zhang, X.; Serra, A.; Goh, B. C.; Darwis, D. A.; Chen, M. W.; Sze, S. K.; Liu, C. F.; Lescar, J.; Tam, J. P. *Proc. Natl. Acad. Sci. U. S. A.* **2019**, *116*, 11737–11746.

(2) Jackson, M. A.; Gilding, E. K.; Shafee, T.; Harris, K. S.; Kaas, Q.; Poon, S.; Yap, K.; Jia, H.; Guarino, R.; Chan, L. Y.; Durek, T.; Anderson, M. A.; Craik, D. J. *Nat. Commun.* **2018**, *2411*.

(3) Harris, K. S.; Durek, T.; Kaas, Q.; Poth, A. G.; Gilding, E. K.; Conlan, B. F.; Saska, I.; Daly, N. L.; van der Weerden, N. L.; Craik, D. J.; Anderson, M. A. *Nat. Commun.* **2015**, *6*, 10199.

(4) Nguyen, G. K. T.; Wang, S.; Qiu, Y.; Hemu, X.; Lian, Y.; Tam, J. P. *Nat. Chem. Biol.* **2014**, *10*, 732–738.

(5) Rajendran, S.; Slazak, B.; Mohotti, S.; Strömstedt, A. A.; Göransson, U.; Hettiarachchi, C. M.; Gunasekera, S. *Phytochemistry* **2021**, *187*, 112749..

(6) Du, J.; Yap, K.; Chan, L. Y.; Rehm, F. B. H.; Looi, F. Y.; Poth, A. G.; Gilding, E. K.; Kaas, Q.; Durek, T.; Craik, D. J. *Nat. Commun.* **2020**, *11* (1575).

(7) Gruber, C. W.; Čemažar, M.; Clark, R. J.; Horibe, T.; Renda, R. F.; Anderson, M. A.; Craik, D. J. *J. Biol. Chem.* **2007**, *282*, 20435–20446.

(8) Burman, R.; Gruber, C. W.; Rizzardi, K.; Herrmann, A.; Craik, D. J.; Gupta, M. P.; Göransson, U. *Phytochemistry* **2010**, *71*, 13–20.
